# Recent Advances on Detection of Insecticides Using Optical Sensors

**DOI:** 10.3390/s21113856

**Published:** 2021-06-03

**Authors:** Nurul Illya Muhamad Fauzi, Yap Wing Fen, Nur Alia Sheh Omar, Hazwani Suhaila Hashim

**Affiliations:** 1 Functional Devices Laboratory, Institute of Advanced Technology, Universiti Putra Malaysia, Serdang 43400, Selangor, Malaysia; illyafauzi97@gmail.com (N.I.M.F.); aliashehomar@gmail.com (N.A.S.O.); 2 Department of Physics, Faculty of Science, Universiti Putra Malaysia, Serdang 43400, Selangor, Malaysia; hazwanisuhaila@gmail.com

**Keywords:** insecticides, optical sensor, recognition element

## Abstract

Insecticides are enormously important to industry requirements and market demands in agriculture. Despite their usefulness, these insecticides can pose a dangerous risk to the safety of food, environment and all living things through various mechanisms of action. Concern about the environmental impact of repeated use of insecticides has prompted many researchers to develop rapid, economical, uncomplicated and user-friendly analytical method for the detection of insecticides. In this regards, optical sensors are considered as favorable methods for insecticides analysis because of their special features including rapid detection time, low cost, easy to use and high selectivity and sensitivity. In this review, current progresses of incorporation between recognition elements and optical sensors for insecticide detection are discussed and evaluated well, by categorizing it based on insecticide chemical classes, including the range of detection and limit of detection. Additionally, this review aims to provide powerful insights to researchers for the future development of optical sensors in the detection of insecticides.

## 1. Introduction

Pesticides are chemical categories that have been designed to protect crops by preventing, destroying, repelling or mitigating any pests [1]. There are many different types of pesticides in industries such as insecticides, rodenticides, herbicides, fungicides, biocides and similar chemicals [2]. They are categorized according to their chemical forms and the types of pests that they kill [3]. The first major synthetic class of pesticides that has been widely used is insecticide [4]. According to United States Environmental Protection Agency, insecticide is commonly used in the agricultural, industrial applications, public health, commercial applications and households. A variety of different insecticides such as organophosphates, carbamates, neonicotinoids, pyrethroids or pyrethrins and organochlorines are classified according to their chemical classes [5].

Today, organophosphates account for around 50% of the chemical pesticides used in controlling the pests. The chemical organophosphates work by disrupting an enzyme in the body called acetylcholinesterases that control the nerve signals in the pest’s body [6]. Carbamates insecticides also play a similar mode as organophosphates. Although they have a similar mechanism of action to that of acetylcholinesterase (AChE) inhibition (phosphorylation by organophosphates and carbamylation by carbamates), organophosphates can bind to AChE irreversibly, meanwhile carbamates bind to AChE reversibly [7]. In addition, the toxicity of carbamates is similar to that of organophosphate pesticides with a duration typically less than 24 h [8]. In addition, neonicotinoids are a fairly new type of insecticide that have been used over the last 20 years to control a variety of pests, especially sap-feeding insects such as cereal aphids and root-feeding grubs. Like nicotine, neonicotinoids work effectively by binding to nerve cell receptors that normally respond to the neurotransmitter acetylcholine. When neonicotinoids over-excite neurons at high doses, it might lead to epileptic symptoms, cell death or inactivation of the nerve cells [9]. In comparison, neonicotinoids are less toxic to birds and mammals compared to organophosphates and carbamates [10]. Subsequently, the uses of pyrethrins have increased rapidly with the declining use of organophosphates, carbamates and neonicotinoids. Pyrethrin is a botanical insecticide derived from chrysanthemum flowers. It became a popular insecticide for the control of agricultural pests. Apart from that, pyrethrin can also fight mosquito-borne diseases. It works by altering nerve function, in the target insect pests which can causes paralysis in, and finally resulting in death. The small quantity used of pyrethrins for the pests controls making them competitively cost-effective [11]. To increase the pyrethrin’s stability on sunlight exposure, the chemical structure of pyrethrin has been modified and it is called pyrethroid [12]. Lastly, organochlorines insecticides have also been used extensively in agriculture and mosquito control. The major difference between pyrethrin and organochlorine is the way it works to kill insects. Organochlorine compounds work on insects by opening the sodium ion channel in insect neurons or nerve cells, spontaneously causing them to spasm, fire and eventually die [13,14].

**Figure 1:**
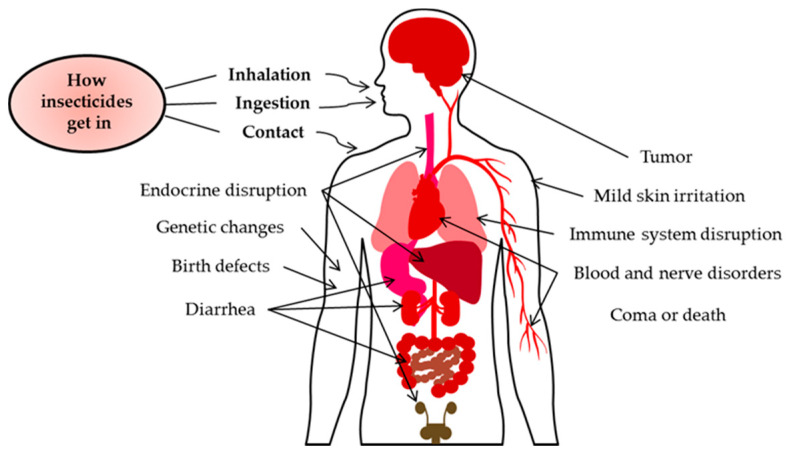
Insecticides’ exposure effects to human health.

Despite insecticide usefulness in many industries, excessive use of it can give a pernicious impact on the environment and ecosystem. It can cause air pollution, water pollution, soil pollution, food and water contamination that will pose a great danger to the human body [15]. This happened because insecticides were secreted into the soils and groundwater that may end up in drinking water. Additionally, the spray of insecticides can drift and pollute the air that can enter the body by inhaling aerosols, dust and vapor, whereas oral exposure to the insecticides can be happened during the consume of food and water or direct contact with the skin [16]. Over time, the chemical will bio-accumulate in the body. The effects of insecticides on human health depend on the toxicity of the chemicals and the exposure period [17]. The simplest examples that have high risk with this toxicity are farm workers and their families. They will experience the greatest exposure to agricultural insecticides through direct contact [18]. The effects from the exposure can be mild skin irritation, tumor, genetic changes, birth defects, diarrhea, blood and nerve disorders, endocrine disruption, immune system disruption, coma or death [16]. Figure 1 briefly shows exposure to insecticides impose danger on humans.

Due to the negative impacts of insecticides, various analytical optical methods for the determination of insecticides were reported in this research literature. These particularly involve optical methods such as fluorescence, colorimetric, surface enhanced Raman scattering (SERS), surface plasmon resonance (SPR), chemiluminescent strategies and more. This review also particularly emphasizes the recognition elements engaged in the analytical methods. Recognition element is also known as target receptor that is in charge of identifying particular analytical targets [19]. Enzymes, antibodies, aptamers and molecularly imprinted polymers (MIPs) are examples of the recognition elements in this literature. A lot of recognition elements have been introduced in the development of optical methods for the detection of insecticides since it is able to enhance the sensitivity of the optical sensors.

## 2. Optical Sensors

Optical sensors are developed based on various technologies of optical phenomena, which are the result of the correlation of an analyte with the receptor part [20]. This may be further subdivided according to the type of optical properties such as reflectance, absorbance, refractive index, fluorescence, luminescence and the light scattering [21]. As there are large numbers of different optical principles that exist, many optical methods have been introduced and are used for a lot of applications. Optical sensors also have proven to be very easy, fast and low-cost approach [22]. For the detection of insecticides, the most favorable optical methods among researchers are fluorescence, colorimetric, SERS, SPR and chemiluminescence. This article focuses on the contact-based optical methods with the recognition elements involved. The comparative analysis of the advantages and disadvantages of these optical techniques had been discussed further by Anas et al. (2018) [23].

### 2.1. Fluorescence

Fluorescence technique is one of the most widely used methods to identify insecticides that are simple to use and have a high sensitivity and selectivity [24]. In addition, it is also widely used for biomedical [25], environmental monitoring [26], food protection and quality control [27,28]. In this method, spectrofluorophotometer can generate the signal shift and be observed by the naked eye on-site [29]. Different types of recognition elements and materials have been used in the manufacturing of fluorescence sensors, including enzymes [30], semiconductor nanomaterials [31], metal nanomaterials [32], carbon materials [33] and noble metal [34], as it can enhance the sensitivity of the sensor.

### 2.2. Colorimetric

The colorimetric sensing technique has been proven to be an efficient analytical sensing of metallic cations, anions, drugs, pesticides, organic dyes and other toxic pollutants due to its easy fabrication [35], high sensitivity and selectivity [36], quick detection [37], as well as easy naked-eye sensing [38]. Since the colorimetric involved quantification of color from the reaction, then converting reaction behavior into visual color change is the key challenge for colorimetric platform manufacturing. Gold nanoparticles (AuNPs) have been widely used as a promising signal transducer in the development of a colorimetric sensor for the detection of insecticides.

### 2.3. Surface Enhanced Raman Scattering

Surface enhanced Raman scattering (SERS) relies on molecules adsorbed on a roughened metallic surface of gold, silver or copper or their nanoparticle to enhance inelastic scattered light. The chemical content of different molecular species can be distinguished by Raman spectroscopy through the collection of molecular vibrations, i.e., Raman spectroscopy [39]. High sensitivity, of the detection coupled with high selectivity in SERS, opens up a wide range of SERS spectroscopy applications such as biomedical diagnosis and environmental monitoring [40,41].

### 2.4. Surface Plasmon Resonance

Surface plasmon resonance (SPR) strategy has drawn great attention as an economical, label-free tool because of its ability to detect target compounds in high sensitivity, real-time manner and rapid detection [42,43,44,45,46,47,48]. The Kretschmann configuration-based SPR method works by detecting the resonance in the form of charge density oscillations between the analyte and a metal thin film [49,50,51,52,53,54,55]. At two dielectric media interfaces, any metal holding a large number of free electrons such as gold, silver, copper and aluminum is positioned [56,57,58]. However, gold is the most stable and sensitive metal compared to others, so it is very suitable to be used as a metal film [59,60,61,62,63,64,65]. SPR will occur under complete conditions of internal reflection when plane-polarized light hits the gold-coated film prism [66,67,68]. Then it will detect the reflected beam for processing. Various types of prepared thin films can give different refractive indices that then will affect the resonance intensity [69,70,71,72,73]. This method has been widely applied in food control, environmental monitoring and drug delivery with outstanding repeatability and reproducibility [74,75,76,77,78,79,80,81]. Due to their superior performance, SPR sensor is gaining more attention in detecting insecticides. Typically, SPR uses antibodies as receptors to catch their target. Enzymes and nanoparticles have also been introduced in enhancing the sensitivity of this sensor.

### 2.5. Chemiluminescence

Chemiluminescence method does not involves light excitation since the energy of a chemical reaction caused by a luminescence reagent is excited by the material [82]. This method have a good sensitivity and specificity, high stability of reagents and their conjugates and is cost-effective, making it an excellent method for the food analysis and diagnosis of disease [83,84]. This method has been successfully used in the identification of insecticides, based on the advantages already described by integrating them with recognition elements such as antibodies, enzymes and nanoparticles.

### 2.6. Others

Apart from the above mentioned methods, there are some other optical methods reported that have been used to detect insecticides, such as electrochemiluminiscence, photoluminescence, phosphorescence, luminescence, liquid chromatography–mass spectrometry (LC-MS/MS), competitive fluorescence-linked immunosorbent assays (cFLISA), enzyme-linked immunosorbent assay (ELISA), lateral flow immunoassay (LFIA) and high fundamental frequency quartz crystal microbalance (HFF-QCM). These methods will also be explained briefly in the literature.

## 3. Recognition Elements

In general, an optical sensor contains a recognition element that can interact specifically with the specific target and the part of the transducer used to signal the binding event [85]. Thus, the selection of the recognition element depends on the target analyte and the recognition element must have a high binding affinity and stability for the target [86]. Recognition elements may be enzymes, antibodies, aptamers and molecularly-imprinted polymers (MIPs) that interact with the analyte to generate a signal using optical sensors.

### 3.1. Enzymes

The enzymatic optical sensors have greatly offer high selectivity and sensitivity for both identification and quantification of target analyte [87]. Mostly, insecticides have been used in the past as the inhibitors of enzyme activity or substrates that play an important role in enzymatic reactions, indirectly inducing the signal response to the optical sensor. As expected, the specificity of enzyme can increase the effectiveness of optical sensor to detect insecticides accurately [88,89]. In this review, the most popular enzymes that have been exploited extensively for the enzymatic detection of insecticides are acetylcholinesterase (AChE) and organophosphate hydrolase (OPH) with few records of other enzymes, such as butyryl cholinesterase (BChE), alkaline phosphatase (ALP), choline oxidase (ChOx), horseradish peroxidase (HRP), tyrosinase (TYR), trypsin (TRY) and streptavidin.

### 3.2. Antibodies

Nowadays, antibodies as bio-recognition elements have been widely used as a potential alternative method for immunoassay for the analysis of insecticides detection [90]. It is also used for the manufacture of immunosensors in the clinical and biochemical sectors [91]. Polyclonal, monoclonal and recombinant antibodies have been frequently used for insecticide detection. Due to the very high equilibrium association constants, these antibodies can recognize antigens sensitively [92,93]. With the development of nanomaterials and nanotechnology, new and wide opportunities have been brought in the development of optical immunosensors with this recognition element.

### 3.3. Aptamers

Aptamer is a single nucleic acid molecule with nucleobases synthesized in vitro without the need for animal or cell culture [94]. Compared to natural antibodies, aptamers are usually simpler and low cost to synthesize as well as having a good stability in sustainable to repetitious renaturation denaturation [95]. Aptamers have a high affinity in the nanomolar to picomolar range to their targets with dissociation constant (Kd) values [96]. In addition, aptamers usually recognize and bind to corresponding targets directly as “lock-and-key” through molecular shape complementarities, stacking of aromatic rings, electrostatic and van der Waals interactions as well as hydrogen bonding, which make their detection much more effective and convenient [97,98].

### 3.4. Molecularly-Imprinted Polymers

Molecularly-imprinted polymers (MIPs) are recognition elements called plastic antibodies with specific recognition capacity [99]. MIPs can be easily prepared by in situ co-polymerization of functional monomers around a template molecule [100]. As a result of its high stability and remarkable mechanical properties, MIPs have also been widely used to improve the efficacy of separation and sensitivity of detection in sensor development [101]. Furthermore, MIPs have great potential to be used as tailor-made polymers in the fabrication of biosensors, especially if the biological recognizers (enzymes and antibodies) are not available. Based on the advantages mentioned, MIPs are gaining attention in the development of drug delivery, biosensor and environmental remediation [102,103].

### 3.5. Others

Apart from the above recognition elements, metal nanomaterials as recognition units and nanoquenchers have also proven to be an attractive approach to improve the performance analysis of insecticide detection. Specific coordination property between metal and insecticide can enhance recognition selectivity, thus provide new insights into the development of insecticide detection systems [104]. Additionally, the introduction of ligand replacement system has also been used for sensitive insecticide analysis in “turn-off-on” mode [105]. Furthermore, some novel fluorescence detection techniques and technologies have been skillfully exploited for insecticide detection based on the direct use of fluorophore as recognition and response elements [106]. This strategy can avoid the modification process, save reaction time and also simplify the detection steps.

## 4. Various Classes of Insecticides Detection by Optical Sensors

**Figure 2:**
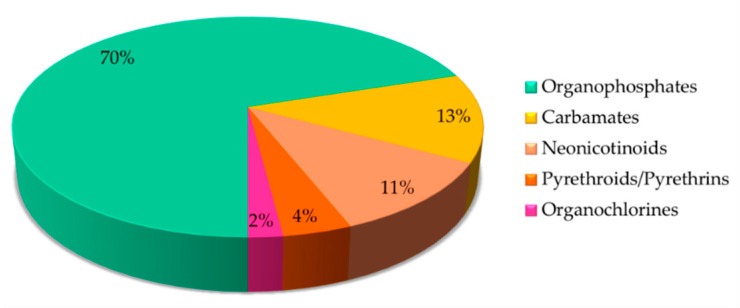
Percentages of various classes of insecticides based on optical sensors.

Insecticides have been commonly used in agriculture, medicine, industry and by consumers over the last few years. The release of untreated effluents from these industries into the ecosystem will lead to the accumulation of toxic insecticides, thus endangering both humans and the environmental [107]. As a result of that, a good detection device such as an optical sensor is essential to identify these insecticide residues in the environment. In this context refer, a well-structured article was written based on optical sensors associated with recognition elements categorized by different insecticides classes. The percentages of various classes of insecticide detection based on optical sensors are summarized in Figure 2.

### 4.1. Organophosphates

**Figure 3:**
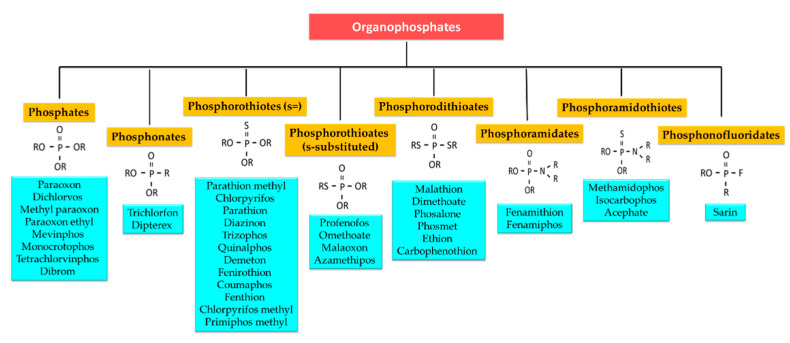
Types of organophosphates based on chemical structure.

Different organophosphates (OPs) compounds have structural similarities within classes. All OPs share one thing in common. They all have a phosphorus atom and a characteristic phosphoryl bond (P = S). Essentially, OPs are esters of phosphoric acid with varying combinations of oxygen, carbon, sulfur or nitrogen attached. For sure, the chemistry of these compounds is much more complex and classification is so confounding. Complexity in classification of OPs arises due to different side chains attached to the phosphorus atom and the position at which the side chains are attached [108]. Throughout this journal, the term organophosphates is used as a generic term to include all the organic compounds containing phosphorus. There are 8 types of OPs detection by optical sensor. Therefore, Figure 3 briefly presents its classes based on side chains and other elements attached to the phosphorus atom consequently.

#### 4.1.1. Phosphates

There are several categories of phosphates that detected by optical sensors such as paraoxon, dichlorvos, methyl paraoxon, paraoxon ethyl, mevinphos, monocrotophos, tetrachlorvinphos and dibrom. All categories have different functions and applications. In medical it can be used as ophthalmologic anti-glaucoma treatment [109,110]. Other applications are to control a broad range of insecticides in agriculture [111,112,113], household [114] and stored product [115,116,117]. For methyl paraoxon, it is oxidization from methyl parathion by solar irradiation and it is very toxic compared to methyl parathion [118].

**Figure 4:**
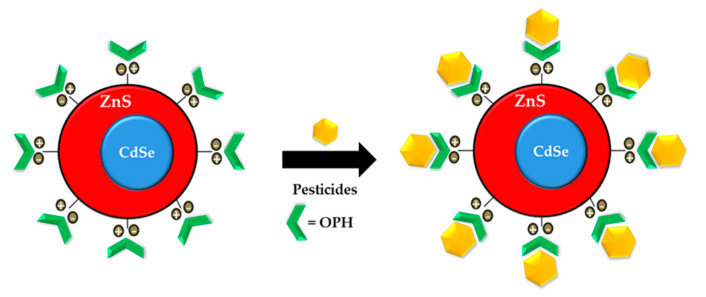
Scheme for the formation of CdSe(ZnS) core-shell QDs-OPH in detection of pesticides [120].

Previous research has shown that paraoxon is one of the first phosphate groups to be involved in an optical sensor-based OPH enzyme. The first work presented by Constantine et al. (2003) fabricated polyelectrolyte architecture and composed the chitosan and OPH polycations along with thioglycolic acid-capped cadmium sellenide-quantum dots (TGA-capped CdSeQDs) as the polyanion. The system works both to detect the presence of paraoxon and to detoxify it by using OPH [119]. The development detection of paraoxon with OPH bioenzyme expanded with cadmium sellenide-zinc sulfide (CdSe(ZnS)) core-shell QDs by Ji et al. in 2005 as a novel biosensor. By electrostatic interaction between negatively charged QDs surfaces and the positively charged protein side chain and ending groups (-NH_2_), the OPH was coupled to (CdSe)ZnS core-shell QDs. Figure 4 is the proposed scheme for the formation of OPH/QDs bioconjugates [120].

**Figure 5:**
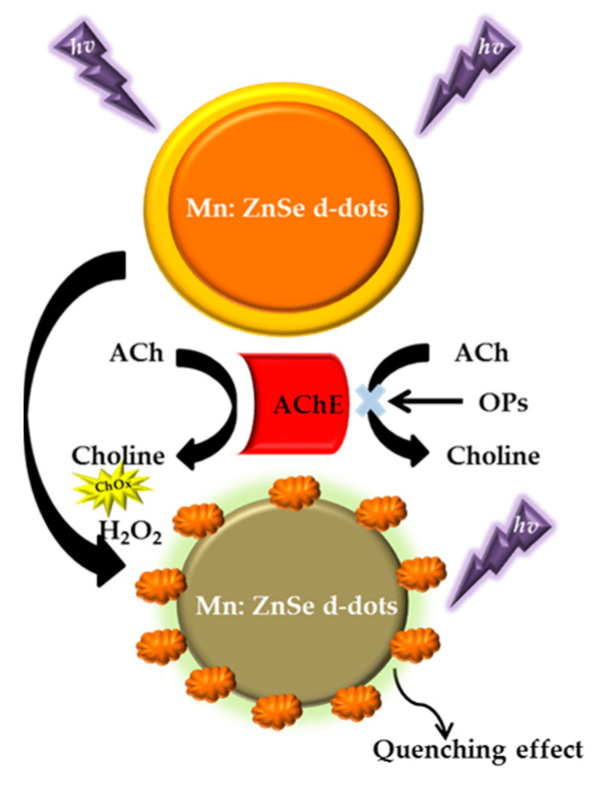
Illustration basic principle of OPs biosensor by AChE enzyme [124].

On the other hand, the detection of paraoxon-based AChE enzymes started in 2009 by Hossain et al. that developed a reagentless bioactive paper-based solid-phase biosensor. The assay strip consisted of AChE paper support and idophenyl acetate (IPA) chromogenic substrate. In this work, malathion, carbaryl and bendiocarb have also been investigated [121]. Another work by Zheng et al. (2011) used the layer by layer (LbL) assembly technique to combine the optical transducer of cadmium tellurite (CdTe) semiconductor QDs with AChE enzymes, resulting in a highly sensitive paraoxon and parathion detection biosensor in vegetables and fruits based on the mechanism of enzyme inhibition [122]. In the following year, Zhang et al. (2012) described the fluorescence method by using AChE to modulate the gap between an AuNPs and the N,N-dimethyldodecylamine N-oxide (DDAO). They found that DDAO is an inhibitor of reversible mixed type-I AChE. DDAO binds to the anionic peripheral site and penetrates through inhibition kinetics test and molecular docking analysis into the active gorge site of AChE. The nanobiosensor has a high sensitivity to tacrine and paraoxon and exhibits different efficiencies of reduction for the two forms of inhibitors as well [123]. In the same year, Gao et al. (2012) reported a sensitive and selective method for the detection of paraoxon based on Mn:ZnSe d-dots-AChE-H_2_O_2_ fluorescence quenching system. In this work, AChE is able to hydrolyze choline into acetycholine (ACh). Subsequently, to produce H_2_O_2_, ChOx oxidizes choline [124,125]. The enzyme-generated H_2_O_2_ can quench the fluorescence of Mn:ZnSe d-dots. Figure 5 depicts the basic operation of an OPs biosensor with AChE enzyme.

**Figure 6:**
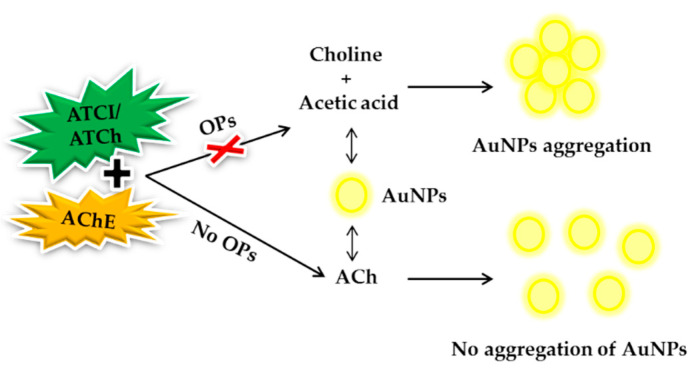
Schematic presentation basic of probing inhibition and reactivation AChE using AuNPs [128].

In the following year, Fu et al. (2013) demonstrated s colorimetric method for OPs detection using Cu(I)-catalyzed click chemistry as the colorimetric signal amplification mechanism between the AChE-ATCl system and azide-terminal alkyne-functionalized AuNPs as the colorimetric probe. In this study, the paraoxon LOD result was obtained at 3.634 nM [126]. Luan et al. (2016) presented fluorescence method and fabricated LbL microarrays of QDs and AChE in the detection of paraoxon and parathion [127]. Subsequently, Wu et al. (2017) synthesized chlorophyll-derived tunable fluorescence emission carbon quantum dots (CQDs). The fluorescence emission can be effectively quenched by AuNPs via fluorescence resonance energy transfer (FRET). Thiocholine, formed by the hydrolysis of BChE from ATCI, might induce the aggregation of AuNPs and the corresponding recovery of the fluorescence emission quenched by FRET. OPs were able to irreversibly inhibit the catalytic activity of BChE, so the recovery effect was minimized. Figure 6 illustrates the schematic of probing inhibition and reactivation AChE using AuNPs [128].

In order to enhance the sensor sensitivity, a label-free bioplatform was developed by Li et al. (2018) for the sensitive detection of OPs via dual-mode (fluorometric and colorimetric) channels based on AChE-controlled quenching of fluorescence CDs [129]. In 2019, Wu et al. fabricated a fluorescence sensor based on BSPOTPE-SiO_2_–MnO_2_ sandwich nanocomposites with AChE enzyme. Thiocholine (TCh) from ATCh by the hydrolysis of AChE, can “turn on” the fluorescence sensor in the detection of paraoxon [130].

Several attempts have been made to detect paraoxon by using other enzymes like eggshell membrane and tyrosinase. In Xue et al.’s work (2016) used CdTeQDs and bi-enzyme-immobilized eggshell membranes for the determination of paraoxon and parathion pesticides. Increasing amounts of OPs have led to a decrease in enzymatic activity and thus to a decrease in H_2_O_2_ production, which is capable of quenching CdTeQDs fluorescence [131]. The modified CdTeQDs by using 5,10,15,20-tetre(4-pyridyl)porphyrin) (TPyP) significantly increased the sensitivity to detect paraoxon [132]. In 2017, Yan et al. developed a fluorescence method for the quantitative detection of OPs via TYR enzyme-controlled quenching of gold nanoclusters (AuNCs). With the presence of OPs, TYR activity was inhibited, resulting in the fluorescence recovery of AuNCs [133].

Another study of phosphate class-based enzymatic detection is dichlorvos. As one of the most popular enzymes, AChE has been exploited extensively by fabricated with QDs based fluorescence method for the diclorvos and other insecticide detection [134,135,136]. Han et al. (2012) proposed a chromogenic platform based on recombinant drosophila melanogaster acetylcholinesterase (R-DmAChE) as the enzyme and indoxyl acetate as the substrate for the rapid study of dichlorvos, omethoate, carbofuran and methomyl. The well-established assay has the capabilities of both qualitative measurements by naked eyes and quantitative analysis by the LOD colorimetric reader of diclorvos at 0.136 μM [137]. In a recent year, Tsagkaris et al. (2020) developed AChE-based LC-MS/MS method to confirm the analytical performance of the assay towards paraoxon, dichlorvos, chlorpyrifos, aldicarb, carbofuran and carbofuran-3 hydroxy [138].

In order to simplify the preparation of the material, Wang et al. (2019) reported QDs without incorporation with any enzyme to detect dichlorvos. In this study, the paper-based fluorescence visualization sensor is produced by combining double QDs with high-activity nanoporphyrins (QDs-nanoporphyrin), and used dichlorvos, demeton and dimethoate in a “turn-off-on” detection mode to achieve specific detection and analysis of OPs [139].

Another study showed that using nanoparticles with AChE enzyme-based colorimetric method in the detection of methyl paraoxon and acephate can provide high sensitivity [140,141]. In the detection methyl paraoxon, Sahub et al. developed the pesticide sensor by graphene quantum dots (GQDs) and AChE with ChOx-based photoluminescence. However, in this approach, the LOD for methyl paraoxon obtained was not as sensitive as the previous study [142].

For the detection of paraoxon-ethyl class using the AChE enzyme, Zhang et al. (2014) demonstrated a novel type of dual emitting probe by using intrinsic dual-emission manganese doped zinc sulfide nanocrystal (ZnS NCs) via turn-on and ratiometric fluorescence. Significantly, the dual-emitting probe had been used to fabricate paper-based test strips for visual detection of paraoxon-ethyl, trichlorfon and chlorpyrifos residues as low as 1.800 µM [143]. Yang et al. (2018) produced from a human plasma sample of 70 μL post-exposures to simultaneously measure both BChE activity and the total amount of BChE (including an inhibited and active enzyme). The idea of this technique is based on the capability of the BChE monoclonal antibody (MAb) to act as both a capture antibody and a detection antibody. The immobilized BChE MAb on the test line was able to identify paraoxon-ethyl [144]. Detection of paraoxon-ethyl by using carbon dots had been investigated by Chang et al. (2017). The fluorescence probe adopted carbon dots as a sensing receptor that has been synthesized in-house via simple acid carbonization of sucrose. This sensing protocol achieved LOD of 0.220 ± 0.020 μM and a dynamic linear range of up to 5.80 mM [145].

The detection for mevinphos and diazinon had been reported by Chen et al. (2010). They developed capillary electrophoresis-laser induced fluorescence (CE/LIF) with QDs. Through the formation of a silane coupling mercaptopropyltrimethoxysilane network, a novel technique to immobilize QDs on the inside capillary surface was created [146]. For the detection of tetrachlorvinphos, Marcos et al. (2014) investigated colorimetric method by immobilized HRP enzyme in a polyacrylamide gel. The HRP-H_2_O_2_ technique was used to measure the pesticides. The sensor can be used for a minimum of 15 days and reacts to tetrachlorvinphos linearly with a detection limit of 0.200 μM [147]. The detection of monocrotophos, methyl parathion and dimethoate was reported in the same year. Long et al. (2014) developed novel nanosensor between NaYF4:Yb,Er UCNPs and AuNPs for monocrotophos and methyl parathion pesticides based on FRET. The detection mechanism is based on the fact that AuNPs inhibit the activity of AChE, which catalyzes hydrolysis of ATC into TCH, by quenching the fluorescence of UCNPs and OPs. In this research, the detection limit of monocrotophos was obtained at 10.305 nM [148].

As expected, by integrating enzyme specificity, great success was achieved in fabricating colorimetric and fluorescence sensors for highly accurate OPs detection. As one of the most popular enzymes, AChE has been exploited extensively for the enzymatic detection of phosphates since 2009. The chronological order of optical sensors for phosphates detection, together with their sensing performance is summarized in Table 1.

#### 4.1.2. Phosphonates

Triclorfon and dipterex are part of the phosphonates family that can be detected by optical sensors. Trichlorfon is used for control cockroaches, crickets, silverfish, bedbugs, fleas, cattle grubs, flies, ticks, leaf miners and leaf hoppers [149]. Dipterex is effective in monitoring leaf eating insects and fruit flies. Therefore, trichlorfon and dipterex come in wettable powder to be used as a foliar spray on vegetables and ornamental sorers against insects and leaf eating caterpillars [150].

Initially, the development of phosphonates detection in trichlorfon started in 2014 [143]. Then, in the following year, He et al. (2015) developed a nanoparticle-based chemiluminescence sensor array for the detection of dipterex. This chemiluminescence sensor array is focused on the simultaneous use of the triple-channel properties of the chemiluminescence-intensive luminol-functionalized silver nanoparticle (Lum-AgNPs) and H_2_O_2_ chemiluminescence system. The chemiluminescence sensor array could identify dipterex and chlorpyrifos as low as 80.134 µM and 68.456 µM, respectively [151].

Subsequently, Trichlorfon detection was got attention from Shen et al. (2016) by using the AChE enzyme. Shen and co-workers developed a new fluorescent probe with 1, 8-naphthalimide dye, quaternary ammonium salt with a boronate group that is water-soluble. The detection assay consisted of the probe, ChOx and AChE, which requires catalyzing ACh by ChOx and AChE to generate H_2_O_2_ and increase the sensitivity of the fluorescence probe. The probe displays the LOD for trichlorfon, methyl parathion and acephate were 0.018 nM, 1277 pM and 0.066 nM, respectively [152].

In a recent year, Dowgiallo et al. (2019) reported the SERS method coupled with colloidal AuNPs. The results presented indicate that this method is a potentially useful tool for identifying trichlorfon, chlorpyrifos, phosmet, coumaphos, methomyl, carbofuran, permethrin and transfluthrin with high sensitivity [153]. Further studies sensors for phosphonates detection are summarized in Table 2 with chronologically order.

#### 4.1.3. Phosphorothiotes (s =)

Optical detection of the phosphorothiotes (s =) groups can be divided into 12 categories: methyl parathion, chlorpyrifos, parathion, diazinon, trizophos, quinalphos, demeton, fenirothion, coumaphos, fenthion, chlorpyrifos methyl and primiphos methyl. These groups are used to control insects and destroy a broad range of pests, especially in agriculture and crops such as fruits [154,155], vegetables [156,157,158] and flowers [159,160]. It is also used to protect household [161] and livestock [162] from insects, act as acarida [163], against ectoparasites in mammalian [164] and as Triatoma control (insects that involved in the transmission of Chagas disease in the Americas) [165].

**Figure 7:**
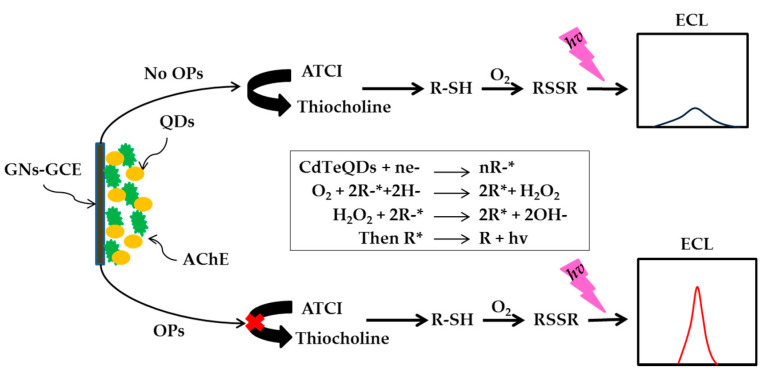
Schematic illustration for the principal of signal-on ECL-biosensor for determination of OPs by using AChE-QDs-GNS-GCE [168].

There are a large volume of published studies describing the optical detection of phosphorothiotes (s =) especially in the detection of methyl parathion. Many studies demonstrated that the use of AChE enzyme-based materials can enhance the sensitivity of the optical sensor. In the work reported by Tran et al. (2012), the fabrication of fluorescence biosensors for pesticide detection was developed from the CdSe, CdTeQDs, AChE and ATCh. The activity of enzyme is directly inhibited by pesticides and able to detect methyl parathion at lower concentration [166]. In the same year, Hai et al. (2012) reported the development of AChE enzyme composed of QDs to detect pesticides conjugated together with MIPs. ATCh was used in this biosensor as an indicator for the function of the AChE enzymes, as it is a very powerful hydrolyte with AChE enzymes present [167]. In the following year, a new highly sensitive and selective electrochemiluminescence assay biosensor based on target-induced signal for OPs detection was developed by Liang et al. (2013), whereby the intelligent integration of graphene nanosheets (GNs), CdTeQDs and AChE enzymatic reaction produces a hybrid biofunctional AChE-GNs-QDs as cathodic ECL emitters for OPs sensing. The detection limit was found to be as low as 0.228 nM [168]. Figure 7 shows the illustration for the principal of signal on electrochemiluminescence biosensor for determination of OPs by using AChE-QDs-GNS-GCE. The LOD was further lowered down to 0.039 pm by enzymatic reaction of self-assembly modulation of gold nanorods (AuNRs) to incorporate with colorimetric assays [169].

**Figure 8:**
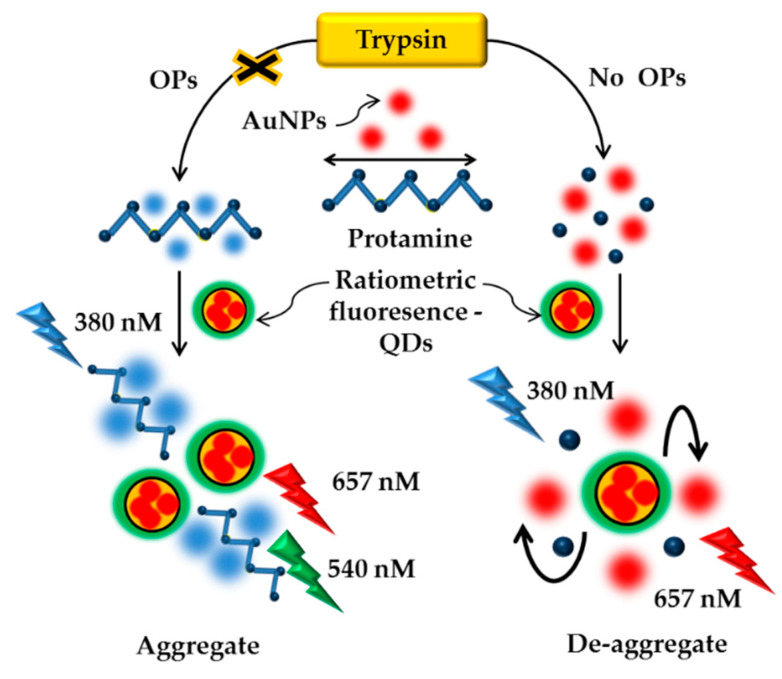
Illustration of the fluorescent detection of OPs through the inner-filter effect of gold nanoparticles on RF-QDs-TRY [171].

Several studies have been published to describe the identification of methyl parathion using OPH, trypsin and HRP/ALP enzymes. Yan et al. (2014) constructed a sensitive fluorescence probing strategy for methyl parathion detection based on ET between p-nitrophenol and CdTeQDs in cetyltrimethylammonium bromide (CTAB) by using OPH enzyme [170]. In order to enhance the sensor sensitivity, Yan et al. (2015) reported a novel fluorescence sensor using trypsin (TRY) enzyme to detect methyl parathion based on the IFE between AuNPs and radiometric fluorescence-quantum dots (RF-QDs). The inhibition efficiency of methyl parathion for trypsin activity was evaluated as low as 68.386 nM by measuring the fluorescence of RF-QDs [171]. Figure 8 shows the fluorescent detection of OPs through the inner-filter effect of gold nanoparticles on RF-QDs-TRY.

**Figure 9:**
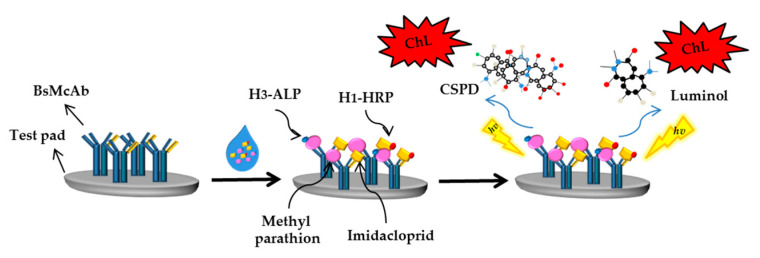
Illustration the chemiluminescence reaction kinetics-resolved MIA strategy for methyl parathion and imidacloprid detection [173].

In 2017, Shu et al. prepared a novel bifunctional antibody (BfAb) that could identify methyl parathion and imidacloprid via hybrid hybridomas technique [172]. For the quantitative detection of pesticide residues, a multiplexed immunochromatographic test strip based on a time-resolved chemiluminescence strategy was developed using BfAb as the sole recognition reagent. HRP and ALP were used as the chemiluminescence probes to label the pesticide haptens as the proposed method. The two chemiluminescence reactions catalyzed by the enzymes were activated simultaneously by injection of coreactants after the labelled haptens competed with pesticides to bind with the BfAb immobilized on the test strip [173]. The chemiluminescence reaction kinetics-resolved MIA strategy for methyl parathion and imidacloprid detection is presented in Figure 9.

**Figure 10:**
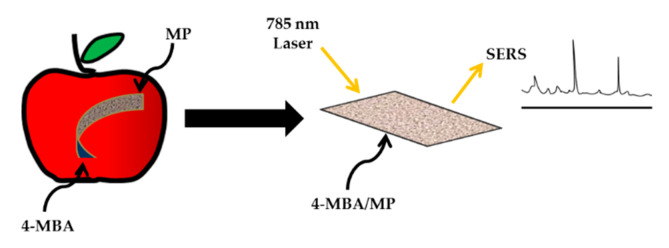
Schematic of paper-based SERS substrate for detecting methyl parathion on the fruit peel surface [179].

A simple and sensitive fluorescent sensor based on L-tyrosine methyl ester functionalized carbon dots (Tyr-CDs) and tyrosinase system was developed in 2015 by Hou et al. for the detection of methyl parathion. The LOD was obtained at 0.048 nM [174]. In the same year, Yan et al. (2015) synthesized fluorescence probe based on near-infrared CuInS_2_-QDs and Pb^2+^ for methyl parathion detection. Due to the competitive binding of Pb^2+^ and mercaptopropionic acid to QDs, the fluorescence intensity of copper indium sulfide (CuInS_2_)-QDs had been quenched in the presence of Pb^2+^ [175]. In 2017, Kashani et al. proposed fluorescence method by using molybdenum disulfide (MoS_2_)-QDs [176]. Metal nanoparticles-based SERS method has also been explored [177,178]. AuNPs with excellent reproducibility and stability were used to generate the substrate. The paper-based substrate was then applied to detect the standard solution of methyl parathion on apple, whose detection limit was down to 0.004 μM. Figure 10 shows a schematic of paper-based SERS substrate on the fruit peel surface [179].

Another study showed that the AChE enzyme is appropriate in the detection of chlorpyrifos. However, this work has not been much reported, as studies using enzymes in the optical detection of chlorpyrifos are still in their early stages. In 2018, Xie et al. developed graphitic carbon nitride (g-C_3_N_4_) as a fluorescent probe and AuNPs as a colorimetric probe [180].

Throughout 2010, QDs have been widely used to detect chlorpyrifos. Zou et al. (2010) described a portable fluorescent sensor that integrates an immunochromatographic test strip assay (ITSA) with a QDs label and a test strip reader for biomonitoring of chlorpyrifos. In this work, 3,5,6-trichloropyridinol (TCP) is used to demonstrate the immunosensor’s performance as a model analyte [181]. Next, Zhang et al. (2010) reported CdTeQDs surface coordination-originated FRET and a basic ligand-replacement turn-on mechanism for highly sensitive and selective chlorpyrifos pesticide detection [182].

**Figure 11:**
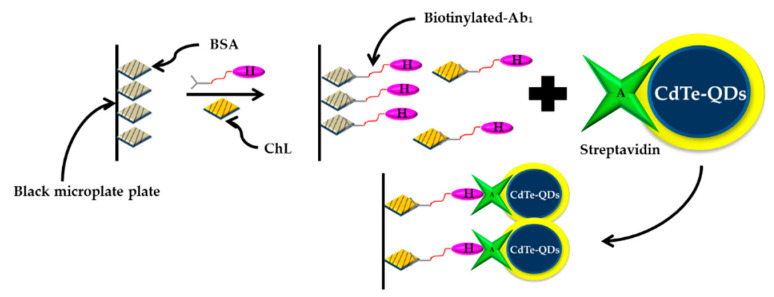
The schematic diagram of the QDs-SA-cFLISA method procedure [184].

In the same year, Chen et al. (2010) reported cFLISA method based on QDs as the fluorescence label coupled with Ab_2_ for the detection of chlorpyrifos in drinking water [183]. However, Chen et al. (2010) modified the development of cFLISA based on QDs-streptavidin (SA). In order to improve the sensitivity of QDs-SA-cFLISA, 3-mercaptopropyl acid stabilized CdTeQDs and SA made via the active ester process. The work significantly increased the sensitivity [184]. The schematic diagram of the QDs-SA-cFLISA method procedure in this research is shown in Figure 11.

**Figure 12:**
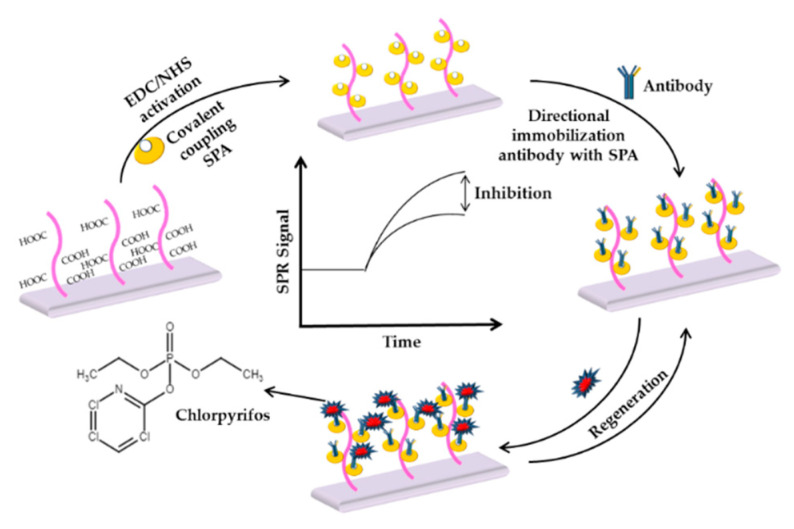
Illustration of experimental procedure [188].

A previous study also reported the use of antibody and MIPs in the detection of chlorpyrifos-based SPR method. By using a two-channel SPR biosensor, Mauriz et al. (2007) performed multi-analyte detection of pesticides which in this design enables chlorpyrifos, carbaryl and DDT to be determined through different formats of immobilization by using each monoclonal antibody [185]. Yao et al. (2013) reported magnetic-MIPs nanoparticles to amplify SPR response and increase the detection sensitivity in SPR spectroscopy [186]. In 2018, Lertvachirapaiboon et al. demonstrated chlorpyrifos detection using an SPR-enhanced photoelectrochemical sensing system [187]. Recently, an SPR biosensor based on an oriented antibody assembly was reported by Li et al. (2019) for the rapid detection of chlorpyrifos residue in agricultural samples. In this study, Staphylococcal protein A (SPA) was covalently bounded to the sensor surface with subsequent binding through its Fc region of the antibody in an oriented fashion. The experimental procedure in this analysis is shown in Figure 12 [188].

The expansion of chlorpyrifos detection in optical sensors had also been developed with the use of metal nanoparticles [151]. In chlorpyrifos analysis, popular trend by using metal nanoparticles-based SERS method has received a lot of attention from researchers [189,190,191]. This method showed potential for on-site environmental monitoring applications. However, due to the competitive adsorption substrates that occur when multiple analytes are present, SERS studies have been limited to detect fewer than five pesticides simultaneously per time [153].

In 2018, Ouyang et al. synthesized g-C_3_N_4_/BiFeO_3_-NCs by a facile one step sol-gel combustion method and employed as a peroxidase-like catalyst. The nanocomposites were used as a colorimetric-chemiluminescent dual-readout immunochromatographic assay (ICA) for the multiplexed detection of chlorpyrifos and carbaryl residues based on the catalytic activity on the luminol-H_2_O_2_ reaction. The LOD of 0.930 nM was obtained [192].

Parathion is another phosphorothioate (s =) that attracts the attention of researchers. Few researchers have reported on the identification of parathion using the enzyme based QDs [127,131,136]. The work presented by Zheng et al. (2011) integrated the optical transducer of CdTe semiconductor QDs with the AChE and phenylalanine hydroxylase (PAH) enzyme by the LbL assembly technique. The LOD obtained was 0.011 nM [122]. In order to enhance the sensitivity, Zheng et al. (2011) modified a nanostructured biosensor by AChE and ChOx enzymes with the same technique. The LOD has been improved down to 0.001 nM [134].

Detection parathion by using antibody had been reported by Kumar et al. (2016). They explored the feasibility of the nanocrystal metal organic framework [Cd(atc)(H_2_O)_2_]n (NMOF1) as a biosensor for the specific recognition of parathion. The luminescence of the NMOF1/anti-parathion complex then was tested for parathion detection. The results showed that effective and stable anti-parathion bioconjugate signals by parathion had an effect on its selectivity and sensitivity [193].

**Figure 13:**
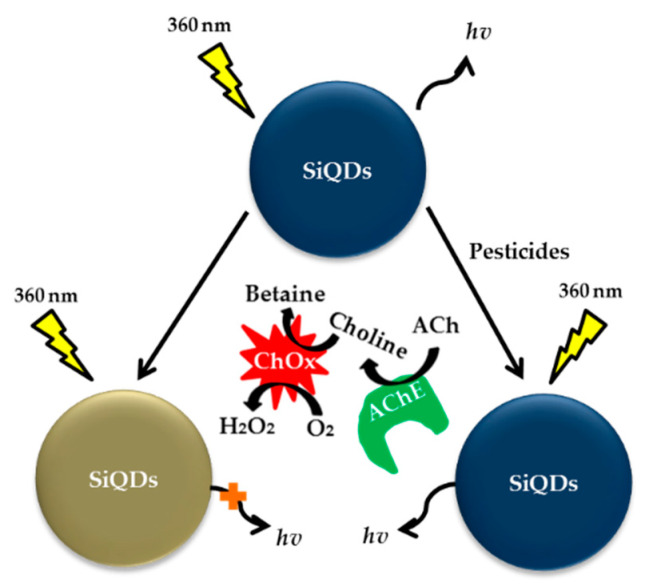
Illustration SiQDs-based sensor for detection of pesticides [195].

In the subsequent years, Zhao et al. (2012) prepared QDs-based MIPs composite nanospheres via a facile and versatile ultrasonication-assisted encapsulation method. The QDs MIP nanospheres were successfully applied to the direct fluorescence quantification of diazinon on the basis of fluorescence quenching through template analytes (diazinon) rebinding into the recognition cavities in the polymer matrixes. This novel technique can selectively and sensitively detect diazinon in water down to 0.164 μM [194]. In the following year, the detection of diazinon was expanded using the AChE enzyme by Yi et al. (2013). In this work, a novel label-free SiQDs-based photoluminescence (PL) sensor was developed for ultrasensitive parathion, diazinon and carbaryl detection. The LOD obtained for parathion and diazinon were 0.112 nM and 0.222 nM, respectively [195]. Figure 13 shows SiQDs-based sensor for detection of pesticides.

On the other hand, Chang et al. (2016) proposed AChE activity in the detection diazinon. They investigated a simple paper-based fluorescence sensor (PFS) based on the aggregation induced emission (AIE) effect of tetraphenylethylene (TPE) and the addition reaction capability of maleimide. Meanwhile, the AChE activity and OPs were obtained, respectively [196]. In a recent year, Wang et al. (2019) developed a highly sensitive upconversion fluorescence biosensor for the detection of diazinon based on an AChE modulated fluorescence ‘off-on-off’ strategy. As a result of an energy transfer effect, the luminescence of synthesized UCNPs could be strongly quenched by Cu^2+^. The enzymatic hydrolyzate (thiocholine) could seize Cu^2+^ from the UCNPs-Cu^2+^ mixture after the addition of AChE and ATCh, resulting in the quenched fluorescence being activated [197].

**Figure 14:**
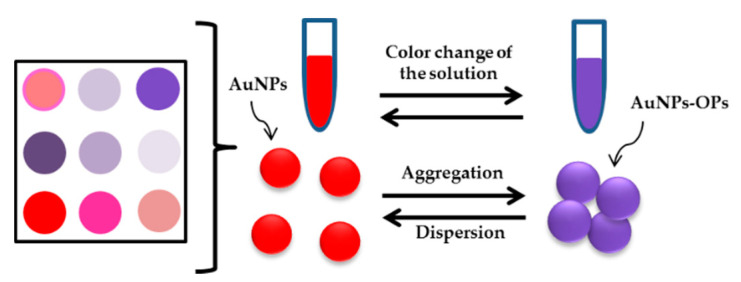
Illustration of AuNPs-based colorimetric sensor in the detection of pesticides [202].

For the detection of trizophos, there have been advancements in the use of antibodies, metal nanoparticles and enzymes-based colorimetric method [198,199]. The combination of antibodies and metal nanoparticles has shown the highest sensitivity and selectivity in detecting trizophos [200]. Other researchers also documented the identification of phosphorothiotes (s =) such as chlorpyrifos methyl, quinalphos, primiphos methyl, demeton and fenirothion [139]. Wang et al. (2014) proposed a novel LFIA focused on three competitive immunoreactions for the simultaneous identification of the pesticides imidacloprid, chlorpyrifos-methyl and isocarbophos. This approach based on the three red channels to detect imidacloprid, chlorpyrifos-methyl and isocarbophos, respectively (three test lines dispensed with various capture reagents). The LOD for chlorpyrifos-methyl was obtained at 0.310 µM [201]. In 2016, the colorimetric sensor array consisting of citrate-capped 13 nm AuNPs was introduced by Kashani et al. to detect and discriminate several OPs such as chlorpyrifos, primophos-methyl, fenamiphos and imidacloprid. With pattern recognition techniques, including hierarchical cluster analysis (HCA) and linear discriminant analysis (LDA), the aggregation induced spectral modifications of AuNPs upon OPs addition had been analyzed. The LOD for chlorpyrifos and primiphos methyl were 0.685 µM and 0.786 µM, respectively [202]. Figure 14 presents the illustration of AuNPs-based colorimetric sensor.

Recently, the SPR study for the identification and determination of the OPs pesticide fenitrothion using an optical fiber sensor was performed by Kant et al. (2020). A thin layer of silver for plasmon generation was deposited on the unclad silica optical fiber core. This was followed by the deposition of a sensing surface comprising a layer of nanoparticles of tantalum(V) oxide sequestered in a rGO nano-scaled matrix. In this investigation, the LOD of 38.000 nM was achieved [203]. Although functionalization of metal nanoparticles for the detection of phosphorothiotes (s =) has been explored and proven to be of potential, their detection capability is still in its early stages. Further studies of phosphorothiotes (s =) detection by optical sensors are presented in chronological framework as shown in Table 3.

#### 4.1.4. Phosphorothioates (S-Substituted)

As for now, there are 4 classes of phosphorothioates (s-substituted) that can be detected using optical sensors, i.e., omethoate, prefonofos, malaoxon and azamethiphos. All of these have separable basic functions in protecting variety of crops and home garden from insects [204,205,206]. It also can be used as antiparasitic medicine in fish farming [207] and in buildings, stores and warehouses to control flies and cockroaches [208,209].

Detection of omethoate has been extensively studied using optical sensors since 2012 [137]. Dou et al. then developed a fluorescence method by using gold-based nanobeacon probe for the first time in 2015 to detect omethoate and isocarbophos pesticides [210]. In 2020, Zhang and the team found a colorimetric sensor to detect omethoate with the concentration of 0.390 nM based on ALP-induced silver metallization on the surface of gold nanorods (AuNRs) [211].

For prefonofos detection, Dong et al. (2012) developed an SPR sensor by using MIP ultrathin films as sensing material and anchored on a gold chip by surface-initiated radical polymerization [212]. The gold surface was first modified by 11-mercaptoundecanoic acid to form self-assembled monolayer (SAM). The LOD obtained was 0.964 pM [213]. Optical detection of profenofos has been expanded with omethoate detection in 2016. Tang et al. (2016) proposed the fluorescence by synthesized CdTe/CdS core–shell QDs with broad-specificity DNA aptamers. In this study, QDs was first conjugated by an amidation reaction with the AMO, which is partly complementary to profenofos, omethoate and isocarbophos pesticides DNA aptamer. Then the DNA aptamer was incubated with QDs-labeled amino-modified oligonucleotide (QDs-AMO) to form duplex QDs-AMO-aptamer. The LODs for profenofos and omethoate were 0.100 μM and 0.230 μM, respectively [214]. The latest study in 2020 by Abdelhameed et al. presented MOFs as an excellent material for chemical species sensors. Eu-IRMOF-3-EBA was built up via post-synthetic modification of IRMOF-3 with ethylbenzoylacetate followed by coordination with Eu^3+^ ions, and the LOD has been successfully lowered down to 0.002 nM [215].

The optical detection of azamethiphos has been investigated by Bhasin et al. recently (2020) through fluorescence technique where the rather higher LOD of 50.000 µM was achieved [216]. The phosphorothioates (s-substituted) detection by the optical sensors presented in Table 4 is arranged in chronological order.

#### 4.1.5. Phosphorodithioates

This section includes several examples of phosphorodithioates class such as malathion, dimethoate, phosmet, ethion, posalone, carbophenothion, azinphos methyl, ethoprophos and methidathion. Randomly, the application for all these insecticides are used for mosquito control [217], control a variety of insects that attack fruits, vegetables, landscaping plants and shrubs and also act as acarida [218,219]. It also can be used on pets to control ticks and insects, such as fleas and ants [220,221,222,223,224,225,226,227,228].

The research for this class has been started back in 2015 where Meng et al. reported a colorimetric method based on the irreversible inhibition of AChE activity to detect malathion with the sensitivity of 0.303 µM [229]. Another attempt by Biswas et al. (2016) used gold nanorods as enzyme mimetics, where a slightly lower sensitivity, i.e., 0.005 mM was obtained [230]. The detection of malathion without involving any enzymes was also started in 2015. Carlos et al. first developed SERS to detect malathion in the peels of tomatoes and Damson plums by multivariate curve resolution, and the LOD obtained was 0.372 µM [231]. Singh et al. (2017) used colorimetric assay with palladium-gold nanorod as nanozyme where the LOD has been lowered to 181.621 nM [232].

For optical detection of phosmet residues, Lina et al. (2017) synthesized a PDs-Ab probe by coupling phosmet antibody with PDs based on poly [2-methoxy-5-(2-ethylhexyloxy)-1, 4-(1-cyanovinylene-1, 4-phenylene)] to obtain the LOD of 0.126 nM [233]. Cakir et al. (2019) investigated SPR sensor chip nanofilms using MIPs of P(EGDMA-MATrp) for the detection of dimethoate with concentration as low as 0.037 nM [234]. The chronological development of phosphorodithioates detection by optical sensors is presented in Table 5.

#### 4.1.6. Phosphoramidates

Phosphoramidates are used to control a wide variety of nematode (round worm) pests [235]. Nematodes can live as parasites on or within a plant. They may be free living or associated with cyst and root-knot formations in plants [236]. Fenamithion and fenamiphos are the types of phosphoramidates that can be detected using optical sensors [202]. The optical detection work was started in 2009, where Qu et al. developed fluorescence spectroscopic technique using supramolecular nano-sensitizers combining of CdTeQDs and p-sulfonatocalix[4]arene [237]. Then, Cui et al. (2011) produced rhodamine B (RB) modified RB-AgNPs-based fluorescence and colorimetric probe to detect fenamithion with a better LOD of 10.000 nM [238]. Chronological phosphoramidates detection by optical sensors is tabulated in Table 6.

#### 4.1.7. Phosphoramidothioates

The classes of phosphoramidothioates that can be detected by optical sensors are methamidophos, isocarbophos and acephate [136,201,210,214]. They are highly active, systemic insecticide/acaricide/avicide residual OPs with contact and stomach effect [239]. The applications are to control a variety of leaf-eating and soil insects in crops. It is capable of managing different forms of pests such as aphids, spider mite, borers and rollers [240,241]. The mode of action this insecticide in insects and mammals are to reduce the activity of necessary enzyme for the functioning of the nervous system called AChE [242]. Most of the sensing systems discussed so far have been based on this enzyme because its ability to recognize insecticides molecules by inhibited the enzyme activity with present of insecticides. Its effectiveness is proven in detecting phosphoramidothioates with the lowest LOD [152]. The chronological details of phosphoramidothioates detection by optical sensors are presented in Table 7.

#### 4.1.8. Phosphonofluoridates

In phosphonofluoridates, only sarin has been reported to be detected by optical sensor. It is a chemical warfare agent and it is known as a nerve agent, which is the most dangerous and fast acting nerve agents [243]. They are similar to OPs insecticides in terms of how they act and what kind of harmful effects they cause [244]. Sarin is also known as GB [245]. Detection of sarin by using the AChE enzyme expanded with the detection of soman and paraoxon [140]. Sun et al. (2011) developed a colorimetric sensing system based on the catalytic reaction of AChE and the aggregation of LA capped AuNPs for OPs nerve agents. In this technique, the LOD for soman, sarin and paraoxon in a spiked fruit sample were obtained as low as 15.000 pM, 28.200 pM and 0.452 mM [246]. Like previously mentioned, enzyme-based sensors can also be conjugated with other support platforms such as QDs [122], fluorophore dye [123] and graphitic carbon nitride [192]. Phosphonofluoridates detection by optical sensor chronologically is presented in Table 8.

### 4.2. Carbamates

Classification of pesticides by carbamates (CMs) is simpler as compared with organophosphates (OPs). Since certain CMs have structural similarities to the neurotransmitter ACh and thus induce direct stimulation of ACh receptors in addition to the inactivation of AChE. Carbamates are considered to be safer than OPs insecticides that irreversibly inhibit AChE that can cause more severe cholinergic poisoning [108]. However, CMs and OPs insecticides are frequently used in combination, with the goal of achieving synergistic interaction and controlling a wide range of insects, including those resistant. Hence, exposure to numerous pesticides for the ecosystem as well as humans and animals is inevitable [247]. The application for carbamates is to destroy infectious or ingested insects [248,249], mites and nematodes in variety of crop [250,251]. It also can used to control aphids, thrips, midges, mosquitoes, larvae, soil insects, spider mites in ornamentals, fruits, vines and grasslands [252,253,254,255].

**Figure 15:**
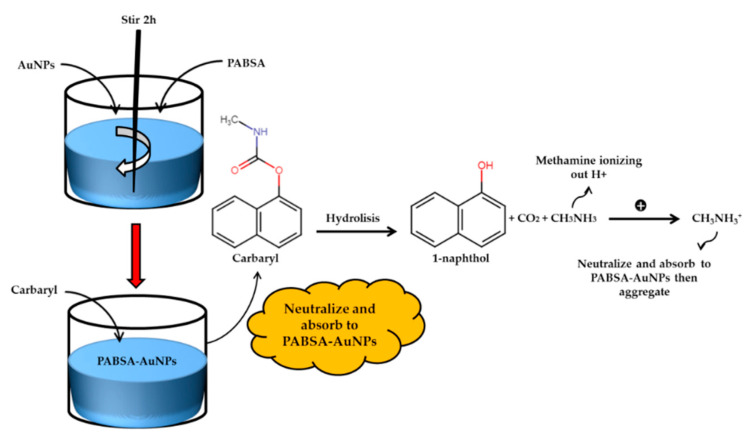
Representation of the carbaryl sensor based on PABSA-AuNPs [259].

Optical detection of carbamates started with detection of methomyl classes in 2007. Li et al. developed luminescent and stable CdTeQDs in sol-gel-derived composite silica spheres and coated with 5,11,17,23-tetra-tert-butyl-25,27-diethoxy-26,28-dihydroxycalix [4]arene(C[4]/SiO2/CdTe) via the sol-gel technique in aqueous media and the LOD obtained was 0.08 μM [256]. For carbofuran, it started in 2009 where Guo et al. examined the simultaneous detection of carbofuran and triazophos with two gold-based lateral-flow strips (strip A and strip B). This study showed that the LOD for carbofuran and triazophos were 32.000 μM and 4.000 μM, respectively [257]. The optical detection of carbaryl started in 2005. Mauriz et al. studied carbaryl using a portable immunosensor based on SPR technology in natural water samples. The assay was based SAM immobilized with monoclonal antibody. The detection limits obtained was 6.858 mM [258]. The detection of carbaryl has been further developed by Sun et al. (2013) where they combined the intriguing optical properties with the inherent zeta potential induced instability properties of p-amino benzenesulfonic acid (PABSA)-AuNPs, based on colorimetric method for detection of carbaryl. The LOD was successfully lowered to 0.250 µM. Figure 15 represents the illustration of carbaryl sensor based on PABSA-AuNPs [259].

A few years later, Zhang et al. (2015) developed a fluorescence sensor based on QDs and with specific recognition for CdSe/ZnS QDs@MIPs. The method developed was simple and efficient for detecting carbaryl with a detection limit of 0.147 μM [260]. Recently, Chiner et al. (2020) developed piezoelectric immunosensors based on high fundamental frequency quartz crystal microbalance (HFF-QCM) for detection of carbaryl and DDT in honey. The biorecognition was based on competitive immunoassays using monoclonal antibodies as specific immunoreagents in the conjugate-coated format. The LOD obtained was 0.248 nM [261]. Shahdost-farda et al. (2020) established a fluorescence method for the detection of carbaryl in Iranian apple using CdTeQDs nanoprobe with the LOD of 0.596 nM [262]. Another biosensor based on the fluorescence approach for determining carbaryl was also reported in 2020. In this study, the researchers prepared B, N-doped CQDs by hydrothermal method. It was found that the florescence of CQDs could be effectively quenched by AuNPs. The fluorescence response with the LOD obtained was 0.298 nM [263]. In the same period, Minh and co-workers constructed a biosensor based on colorimetry to determine carbaryl. The research involved synthetization of Ag@rGO by a simple photochemical process with the GO nanosheets as both stabilizing and reducing agent. This system could susceptibly detect carbaryl with the lowest concentration of 42.000 nM [264].

**Figure 16:**
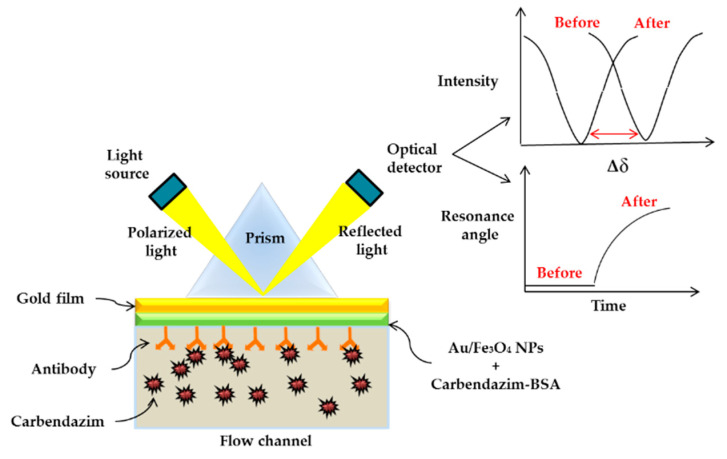
Illustration of the principle SPR technology by Au/Fe_3_O_4_ nanocomposites coupled with antibody [267].

There were only a few studies of detection for metolcarb, aldicarb and carbendazim. Zeng et al. (2015) synthesized NOC_4_ by clicking on a microstructured Au surface and using contact angle measurements to exhibit selective macroscopic recognition of metolcarb. In this study, the LOD was obtained for metolcarb at 0.100 µM [265]. In 2019, Chen et al. synthesized AuNPs-based SERS methods for the detection and quantification of carbendazim in Oolong tea with the LOD of 0.523 µM [266]. Consequently, Li et al. (2019) investigated an SPR biosensor for the carbendazim using Au/Fe_3_O_4_ nanocomposite as an amplifying label on the surface the carboxymethyldextran-coated gold layer of the sensor. The surface was further modified with a monoclonal antibody to detect carbendazim. Immobilized Au/Fe_3_O_4_ nanocomposites on the SPR biosensor enhance the SPR curve through an intensity change, which increases the sensitivity down to 2.301 nM. Figure 16 shows the illustration of the principle SPR technology by Au/Fe_3_O_4_ nanocomposites coupled with antibody [267].

However, the use of AChE enzyme in the detection of carbamates is not as much as OPs [121,137,138,195]. Most recently, researchers are exploring the potential of metal nanoparticles, antibodies and MIPs for the development of carbamates detection [151,153,185,192,234]. Chronological timeline of carbamates detection by optical sensors is tabulated in Table 9.

### 4.3. Neonicotinoids

The EPA classifies neonicotinoids as both toxicity class II and class III agents and is labelled with the signal term “Warning” or “Caution.” These insecticides are more specifically toxic to insects than to mammals because neonicotinoids block a specific neuronal pathway that is more prevalent in insects than in warm-blooded animals [268]. Thus, the use of neonicotinoids such as acetamiprid, thiacloprid, imidacloprid and chlorothalonil are increasing throughout the year in controlling insects. Usually neonicotinoid is used in agriculture. It is a wide-spectrum pesticide that can be used on plants, from leafy vegetables and fruit trees to ornamental plants [269]. Specifically it can be used in seed treatment and managing crops disease [270]. Other than that, neonicotinoids can be used to control insects in households and prevent sucking insects on pets [271]. It is an effective element for controlling a wide range of pests that are otherwise difficult to control [272].

Optical detection of Neonicotinoids was started in 2014 for the acetamiprid classes where Weerathunge et al. investigated the colorimetric biosensing assay to integrate with the intrinsic peroxidase-like nanozyme activity of high affinity GNPs [273]. In the following year, Yang et al. developed the heminfunctionalized reduced graphene oxide (hemin-rGO) composites in the colorimetric method. [274]. For the next reporting period, a novel aptamer-based nanosensor was reported by Hu et al. (2016) for the detection of acetamiprid using FRET between NH_2_-NaYF4:Yb, holmium silica dioxide (Ho@SiO_2_) UCNPs and AuNPs [275]. In the same year, Lin et al. (2016) constructed a novel turn-on sensor for quantification and imaging of acetamiprid. The ZnS:Mn-aptamer acetamiprid aptamer-modified probe was obtained by conjugating ZnS:Mn QDs with the acetamiprid binding aptamer. Multi-walled carbon nanotubes (MWCNTs) dependent on FRET between ZnS:Mn-Aptamer and MWCNTs have been switched off by the fluorescence of the probe [276].

**Figure 17:**
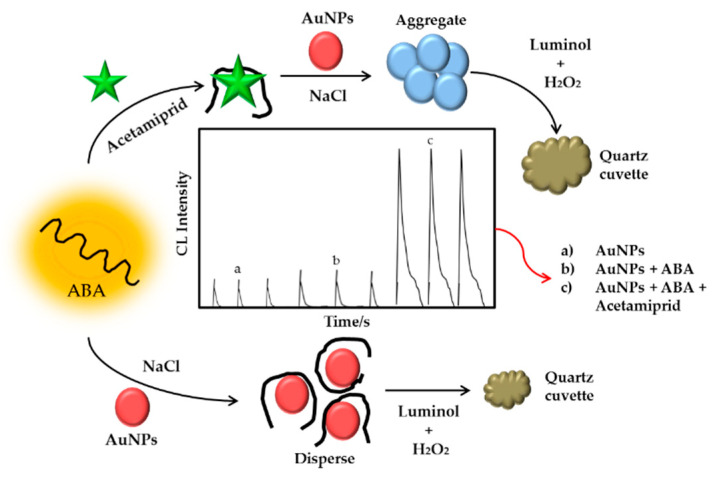
Schematic illustration of the proposed chemiluminescence assay for acetamiprid detection [280].

Several attempts have been made to detect acetamiprid by using AuNPs. Xu et al. (2011) developed a method based on the strong interaction of the cyano group of acetamiprid with AuNPs for the identification of the insecticide acetamiprid [277]. Then, Shi et al. (2013) developed an aptamer-based colorimetric method for highly sensitive acetamiprid detection, taking advantage of the sensitive target-induced color changes that occurred during AuNP aggregation from interparticle plasmon coupling [278]. Next, Yan et al. (2014) investigated the sensing approach based on the inner filter effect (IFE) of AuNPs on RF-QDs for the visual and fluorescent detection of acetamiprid. AuNPs that are based on IFE could quench the photoluminescence intensity of RF-QDs [279]. A few years later, Qi et al. (2016) reported chemiluminescence sensing for detection of acetamiprid based on the high binding affinity of aptamer to target and the relevance between the morphology of AuNPs and its catalytic effect in the presence of H_2_O_2_ and luminol to stimulate chemiluminescence generation. The proposed pesticide residue sensing platform showed a high acetamiprid sensitivity with a detection limit of 62.000 pM [280]. Schematic illustration of the proposed chemiluminescence assay for acetamiprid detection is shown in Figure 17. In the same year, Tian et al. investigated the impact of shortening aptamer sequences on acetamiprid colorimetric detection using aptamer-wrapped AuNPs [281]. In 2020, Qi et al. constructed by the direct and receptive response to the aptamer structure shift induced by acetamiprid of positively charged gold nanoparticles (+) AuNPs [282].

Several studies have revealed the detection of acetamiprid by using AChE and streptavidin. In 2013, Hai et al. presented the new findings of the biosensor made from surface-modified quantum dots of AChE enzymes for optical pesticide detection. In this analysis, CdTe, CdSe/ZnS and CdSe/ZnSe/ZnS-thick shell QDs are new to the QDs described. The findings showed that all the QDs in the biosensor are fit for the position of transducers. Streptavidin-AChE QDs are used in biosensors as a substrate for pesticide detection. Methyl parathion and acetamiprid are the pesticides used in this work. The ATCh is used as an indicator of the activity of the AChE enzyme. Through this study, the LOD was 4.491 nM [283]. Abnous et al. (2016) described the insecticide acetamiprid by an aptamer-based fluorescence. It is based on the target induced release from the aptamer/CS conjugate of the fluorescence in-labelled complementary strand of the aptamer (CS) double stranded DNA (dsDNA). Three types of nanoparticles were used with opposite effects on the fluorophore (FAM). These include Streptavidin-coated AuNPs, single-walled carbon nanotubes (SWNTs) and silica nanoparticles (SiNPs). The assay was highly selective for acetamiprid and has a LOD as low as 127.000 pM [284].

Optical detection of thiacloprid and imidacloprid was began by Li et al. (2014) where they developed a bi-enzyme tracer direct dc-ELISA based on anti-imidacloprid and anti-thiacloprid antibodies. Under the optimized conditions, the LODs for thiacloprid and imidacloprid were obtained at 0.017 µM and 0.008 µM, respectively [285]. In recent year, Tan et al. (2020) applied monoclonal antibody 4D9, colloidal gold (CGN) and time-resolved fluorescence nanobeads (TRFN), respectively, to develop a LFIA for imidacloprid detection in the present work [286]. Lastly, Zhao et al. (2020) proposed a paper-based SERS amplified by virtues of multi-layered plasmonic coupling amplification. The SERS multi-layer was constructed by 3D silver dendrites (SD)/ electropolymerized molecular identifiers (EMIs)/AgNPs sandwich hybrid with multiple hot spots and a strong electromagnetic field in nanogaps. This fabricated SERS paper chips demonstrated impressive specificity and ultrahigh sensitivity in the detection of imidacloprid, with a LOD as low as 0.110 nM [287]. This section demonstrates nanoparticles for neonicotinoids detection-based sensors can be promising and cost efficient techniques. Neonicotinoids detection by optical sensors based on the chronological order is presented in Table 10.

### 4.4. Pyrethroids/Pyrethrins

Synthetic pyrethroids/pyrethrins are commonly used because of their selective insecticidal action, rapid biotransformation and excretion by the class catabolism mechanism and their surrounding environment and the broad-spectrum pest control agents in agricultural production. The use of these insecticides also leads to devastating effects for humans [288]. There are a variety of applications to control a wide range of pests. It is primarily used to handle the various insects and mites that infest fruit plants, vegetables and other crops [289]. They also can regulate plagues include aphids, beetles from Colorado and larvae from butterflies [290]. They also can be used in against cockroaches, fleas and termites in houses and other buildings [291,292,293].

The earliest study in optical detection of pyrethroids/pyrethrins classes is cyhalothrin. In 2010, Li et al. reported the MIPs-based fluorescence nanosensor which is developed by anchoring the MIPs layer on the surface of silica nanospheres embedded CdSeQDs via a surface molecular imprinting process [294]. In 2016, MIPs-SiO_2_-based fluorescence was reported by Wang et al., which could detect λ-cyhalothrin in water samples quickly and effectively [295]. In the same year, Wei et al. developed fluorescence method by using octadecyl-4-vinylbenzyl-dimethyl-ammonium chloride (OVDAC) as a surfactant to transfer aqueous CdTeQDs to detect λ-cyhalothrin [296].

**Figure 18:**
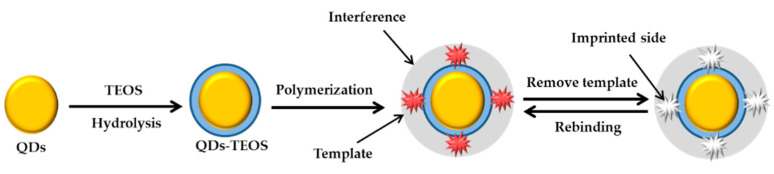
The preparation of MIPs-coated QDs [297].

Previous research has shown that the detection of cyphenothrin was started by Ren et al. (2014). They fabricated MIPs material and successfully utilized it to develop a QDs-based MIPs-coated composite for selective recognition of the template cyphenothrin as highlighted in Figure 18 [297]. On the other hand, Xiaou et al. (2015) demonstrated fluorescence quenching properties of cypermethrin MIPs-QDs. In this analysis as proven that it is possible to use the ELISA approach based on MIPs-QDs to successfully detect residual cypermethrin [298]. In this section, MIPs have been widely reported to be able to detect pyrethins/pyrethroids at high sensitivity and selectivity. However, this technique also has some drawbacks that will discuss further. The details about pyrethroids/pyrethrins detection by optical sensors are presented in Table 11 by chronological order.

### 4.5. Organochlorines

Organochlorines (OC) pesticides are commonly used as synthetic pesticides all over the world. They belong to a group of derivatives of chlorinated hydrocarbons with broad applications in the chemical industry and agriculture. They are acknowledged for their high toxicity, slow degradation and bioaccumulation. Although some of the OC compounds in developing countries have been banned, the use of these agents still increased [299,300]. The application of organochlorine are to fight malaria, typhus and the other insect-borne human diseases [301]. Another application was used to treat scabies and lice. It is also used in agriculture for controlling pest on variety of crops [302,303]. In households, it can be used in mothproofing to clothes and carpets [304]. In the optical detection of OC, antibody is one of the recognition elements that have been selected as reported before for others insecticide detection. The use of this antibody usually required for specificity considerations [185,263,305]. Only Kubackova et al. (2015) identified the OC pesticides aldrin, endosulfan, lindane and dieldrin using SERS by functionalized metal nanoparticles [306]. Chronological range of OC detection by optical sensors to the present day is presented in Table 12.

## 5. Analysis and Conclusions

In the past decades, the wide application of insecticides in the agriculture industry has caused negative impacts in human health through various mechanisms of action. In order to achieve a good solution to this problem, highly sensitive detection of insecticides residues is very important. Based on the review provided, optical sensors such as fluorescence, colorimetric, SERS, SPR and chemiluminescence have attracted tremendous attention from researchers to be used for the detection of insecticides. Refering to the work that has been discussed, the stability and accuracy of this optical sensor can be improved by identifying the appropriate recognition system for the selected analyte. For instance, the bi-enzyme cascade catalytic format has the advantage of multi-signal amplification that greatly enhances the sensitivity of the sensor. In addition, using monoclonal, polyclonal and recombinant antibody against a particular target also can enhance sensitivity and selectivity. Furthermore, in detecting pesticides, MIPs was reported to have advantages as artificial receptors in the QDs and it causes high affinity in the reaction phase. The use of aptamers is also considered to have high stability and sensitivity in identifying insecticides.

Although these optical methods have shown good performance in detecting insecticides when incorporated with recognition elements, there are still many sustainable challenges that need to be tackled in the region. In particular, most of these optical sensors studies remain in laboratory research and are not used in practical applications. The challenges from the surrounding environment such as temperature and pH can affect the stability of the enzyme and antibody during the recognition events that can cause poor and slow reactions. MIPs are also commonly susceptible to matrix interferences and sometimes, the synthesis process and extraction of template molecules are quite complicated. For aptamers, it is often hampered by a sluggish chemical and biological reaction. Furthermore, aptamers require appropriate care to maintain their stability. Therefore, any future projects that will be developed should also concentrate specifically on overcoming the challenges above.

Further studies may try to use metal nanomaterials that are suitable for certain insecticides since they are cheaper, easy to handle and synthesize. In addition, studies on the optical properties and the ability of this composite in the detection of insecticides are very limited and infancy. Corresponding to the conventional method mentioned, SPR proposed an economical, label-free detection method showing ease operation and rapid detection compared to other optical methods. It is envisaged that the metal nanomaterials-based SPR sensing, will exhibit excellent selectivity and sensitivity in detection insecticides from nano to femto. Therefore, insecticide residues in the environment can be determined by this method, providing the potential for broad application in real samples in the future. This approach also prevents the presence of unstable enzymes and complicated chemical modifications or synthesis of antibodies, MIPs and aptamers making it more feasible and cost-effective. For additional information, Table 13 summarizes and compares examples of recognition elements and metal nanoparticles that were used to detect insecticides based on optical methods with the lowest LOD achieved to date.

## Figures and Tables

**Table 1:** Chronological order of optical sensors for phosphates detection.

Type of Phosphates	Method	Material	Range of Detection	LOD	Year	References
Paraoxon	Photoluminescence	chitosan-CdSeQDs/OPH	0–1.000 µM	1.000 µM	2003	[119]
Paraoxon	Photoluminescence	(CdSe)ZnSQDs-OPH	0.010–10.000 µM	0.010 µM	2005	[120]
Paraoxon	Colorimetric	IPA/sol-gel derived silica inks-AChE	0–10.000 µM	1.000 nM	2009	[121]
Mevinphos	Fluorescence	CdTe/CdS coreshell QDs	0.892–124.917 µM	0.714 µM	2010	[146]
Paraoxon	Fluorescence	CdTeQDs-AChE	-	2.750 pM	2011	[134]
Dichlorvos				2.090 pM		
Paraoxon	Fluorescence	PAH/CdTeQDs-AChE	-	0.011 nM	2011	[122]
Dichlorvos	Colorimetric	indoxyl acetate-R-DmAChE	4.525 pM–0.453 µM	0.136 µM	2012	[137]
Paraoxon	Fluorescence	AuNPs/DDAO-AChE	-	0.400 μM	2012	[123]
Paraoxon	Phosphorescence	Mn:ZnSe d-dots–H_2_O_2_-AChE	-	0.013 nM	2012	[124]
Methyl-paraoxon	Colorimetric	Fe_3_O_4_/MNP-AChE/CHOx	-	10.000 nM	2013	[140]
Paraoxon	Colorimetric	ATCl/AuNPs-AChE	3.634–363.376 nM	3.634 nM	2013	[126]
Dichlorvos	Fluorescence	QDs-AChE/ChOx	4.490–6780 nM	4.490 nM	2013	[135]
Tetrachlorvinphos	Colorimetric	oxyferryl-HRP	-	0.200 µM	2014	[147]
Paraoxon-ethyl	Fluorescence	Mn-ZnS nanocrystal/AChE	0–100.000 µM	1.800 µM	2014	[143]
Paraoxon	Phosphorescence	Mn-ZnSQDs/AChE	-	0.100 pM	2014	[125]
Monocrotophos	Fluorescence	NaYF4:Yb,Er/UCNPs-AChE	0.009–89.606 nM	10.305 nM	2015	[148]
Paraoxon	Fluorescence	CdTeQDs	-	4.300 pM	2016	[131]
Methyl-paraoxon	Colorimetric	PAA-CeO_2_/AChE	-	0.108 µM	2016	[141]
Dichlorvos				0.035 M		
Paraoxon	Fluorescence	TPyP-CdTeQDs	9.090 pM–1.090 µM	3.150 pM	2016	[132]
Parathion	Fluorescence	QDs-AChE	-	3.433 nM	2016	[127]
Paraoxon	Fluorescence	QDs-AChE/ChOx	0.001–0.01 µM	0.050 µM	2017	[136]
Dibrom						
Diclorvos						
Paraoxon-ethyl	Fluorescence	carbon dots	-	0.220 ± 0.020 µM	2017	[145]
Paraoxon	Fluorescence	carbon quantum dots-AChE/CHOx	0.182–181.688 nM	0.182 nM	2017	[128]
Paraoxon	Fluorometric	gold nanocrystal-TYR	-	0.363 nM	2017	[133]
Diclorvos	Photoluminescence	graphene quantum dots-AChE/ChOx	0.453–45.253 µM	0.778 µM	2018	[142]
Methyl-paraoxon				0.340 µM		
Paraoxon-ethyl	Colorimetric and fluorometric	monoclonal antibody-BChE	0–7.170 nM	0.100 nM	2018	[144]
Paraoxon	Fluorescence	carbon dots-AChE	0–1.817 nM	0.472 nM	2018	[129]
Diclorvos	Fluorescence	QDs-nanoporphyrin	45.253–90.506 nM	45.253 nM	2019	[139]
Paraoxon	Fluorescence	BSPOTPE-SiO_2_-MnO_2_-AChE	3.634–362.358 nM	3.634 nM	2019	[130]
Paraoxon	LC–MS/MS	AChE	-	5.087 nM	2020	[138]
Diclorvos				23.021 pM		

where LOD is limit of detection. ATCI: azide-terminal alkyne-functionalized, BSPOTPE: 1,2-Bis[4-(3-sulfonatopropoxyl)phenyl]-1,2-diphenylethene, CdS: cadmium sulfide, CdSe: cadmium selenide, CdTe: cadmium tellurite, DDAO: N,N-dimethyldodecylamine N-oxide, CeO_2_: cerium(IV) oxide, LC-MS/MS: Liquid chromatography tandem mass spectrometry, LSPR: localized surface plasmon resonance, Mn: manganese, MNP: magnetic nanoparticles, MnO_2_: manganese(IV) oxide, MOF: metal organic frameworks, IPA: indophenyl acetate, PAA: peroxyacetic acid, PAH: poly(allylamine hydrochloride), R-DmAChE: recombinant Drosophila melanogaster acetylcholinesterase, SiO_2_: silicon dioxide, TPyP: tetra(4-pyridyl)porphyrin, UCNPs: upconventional nanoparticles, ZnS: zinc sulfide.

**Table 2:** Chronological order of optical sensors for phosphonates detection.

Type of Phosphonates	Method	Material	Range of Detection	LOD	Year	References
Trichlorfon	Fluorescence	Mn-doped ZnSNCs/AChE	0–100.000 µM	1.800 µM	2014	[143]
Dipterex	Chemiluminescence	Lum-AgNP	0–80.134 µM	80.134 µM	2015	[151]
Trichlorfon	Fluorescence	1, 8-naphthalimide/AChE-ChOx	-	0.018 nM	2016	[152]
Trichlorfon	SERS	AuNPs	0.388–27.978 µM	3.885 µM	2019	[153]

where LOD is limit of detection. Lum-AgNP: luminol-functionalized silver nanoparticles, Mn: manganese, SERS: surface enhanced Raman scattering, ZnSNCs: zinc sulfide nanocystals.

**Table 3:** Chronological order of optical sensors for phosphorothiotes (s =) detection.

Type of Phosphorothiotes (s =)	Method	Material	Range of Detection	LOD	Year	References
Chlorpyrifos	SPR	monoclonal antibody	0.051–0.154 nM	0.143 nM	2007	[185]
Chlorpyrifos	cFLISA	CdTe/QDs-streptavidin	-	10.839 nM	2010	[183]
Chlorpyrifos	Fluorescence	QDs/TCP	0–2.852 nM	2.852 nM	2010	[181]
Chlorpyrifos	Fluorescence	CdTeQDs	0.100–10.000 nM	0.100 nM	2010	[182]
Chlorpyrifos	cFLISA	QDs/antibody	43.355–586.155 nM	143.187 nM	2010	[184]
Diazinon	Fluorescence	(CdTe/CdS) QDs	0.329–98.571 µM	0.394 µM	2010	[146]
Methyl parathion	SERS	AuNPs/mono-6-thio-b-cyclodextrin with mono-6-thio-b-cyclodextrin	-	0.300 µM	2010	[177]
Parathion	Fluorescence	PAH/CdTeQDs-AChE	0.100–1.000 µM	0.011 nM	2011	[122]
Parathion	Fluorescence	CdTeQDs-ChOx/AChE	-	0.001 nM	2011	[134]
Diazinon	Fluorescence	QDs-MIP	0.164–1.971 µM	0.164 µM	2012	[167]
Methyl parathion	Fluorescence	CdSe/CdTeQDs-AChE	0.190–37.992 µM	0.190 µM	2012	[166]
Methyl parathion	Photoluminescence	CdSe/ZnSe 2MI/ZnS 8MI-QDs-AChE	0.190–30.394 nM	0.190 µM	2012	[137]
Parathion Diazinon	Photoluminescence	SiQDs-AChE/ChOx	25.712 pM–2.571 µM 24.610 pM–2.461 µM	0.112 nM 0.222 nM	2013	[195]
Methyl parathion	Electrochemiluminescence	GNs-CdTe/QDs-AChE	0–0.570 µM	0.228 nM	2013	[168]
Chlorpyrifos	SPR	Fe_3_O_4_@PDA NPs-AChE	0.001–10.000 µM	0.760 nM	2013	[186]
Chlorpyrifos	Fluorescence	Mn-ZnS NCs	0–100.000 µM	1.800 µM	2014	[143]
Methyl parathion	Fluorescence	CdTeQDs/ CTAB-OPH	-	68.386 nM	2014	[170]
Chlorpyrifos-methyl	LFIA	monoclonal antibody	0.310–18.605 µM	0.310 µM	2014	[201]
Methyl parathion	Fluorescence	NaYF4:Yb,Er/UCNPs-AChE	-	2.545 pM	2015	[148]
Methyl parathion	Fluorescence	tyrosinase-carbon dots	0.100 nM–0.100 mM	0.048 nM	2015	[174]
Methyl parathion	Fluorescence	CdTeQDs- Trypsin	-	68.386 pM	2015	[171]
Chlorpyrifos	Chemiluminescent	Lum-AgNP	0–68.456 µM	68.456 µM	2015	[151]
Methyl parathion	Fluorescence	CuInS_2_QDs-Pb^2+^	0.100–38.000 µM	0.060 µM	2015	[175]
Methyl parathion	Chemiluminescence	bispecific monoclonal antibody-HRP/ALP	-	1.254 nM	2015	[172]
Methyl parathion	Colorimetric	gold nanorods-AChE	0.120–40.000 pM	0.039 pM	2015	[169]
Chlorpyrifos Primophos-methyl	Colorimetric	AuNPs	-	0.685 µM 0.786 µM	2016	[202]
Parathion	Fluorescence	CdTeQDs	-	4.300 pM	2016	[131]
Methyl parathion Chlorpyrifos	SERS	AuNPs	0–9.878 nM 0–7.416 nM	9.878 nM 7.416 nM	2016	[189]
Diazinon	Fluorescence	AIE/TPE-maleimide-AChE	0.986–1.643 nM	1.643 nM	2016	[196]
Methyl parathion	Fluorescence	1, 8-naphthalimide-AChE/ChOx	-	1.277 pM	2016	[152]
Trizophos Methyl parathion	Colorimetric	Antibody/AuNPs	-	0.064 nM 3.115 nM	2016	[198]
Parathion	Fluorescence	QDs-AChE	-	3.433 µM	2016	[127]
Parathion	Fluorescence	[Cd(atc)(H_2_O)2]n (NMOF1)-anti parathion	3.433 nM–3.433 µM	3.433 nM	2016	[193]
Trizophos	Colorimetric	GAA-MPA-AuNPs	0.500–500.000 µM	0.080 µM	2016	[199]
Chlorpyrifos	SERS	popcorn like AuNPs	1.000–6.250 µM	1.000 µM	2017	[190]
Methyl parathion	Fluorescence	MOS_2_-QDs	-	0.3229 µM	2017	[176]
Methyl parathion	Chemiluminescent	bifunctional antibody- HRP/ALP	-	0.220 nM	2017	[173]
Parathion	Fluorescence	CdSe/ZnS QDs-AChE/ChOx	-	0.050 µM	2017	[136]
Trizophos	Colorimetric	AuNPs /mcAbs	-	0.045 nM	2017	[200]
Chlorpyrifos	Fluorescence	g-C_3_N_4_/AuNPs-AChE	-	6.900 pM	2018	[180]
Chlorpyrifos	Colorimetric and chemiluminescent	dual-g-C_3_N_4_/BiFeO_3_	-	0.930 nM	2018	[192]
Chlorpyrifos	SPR	TiO_2_/P3HT/AuNPs	0.010–16.000 µM	7.500 nM	2018	[187]
Chlorpyrifos	SPR	staphylococcal protein A	0.713–142.617 nM	15.973 nM	2019	[188]
Chlorpyrifos Coumaphos	SERS	AuNPs	0.003–28.523 µM 0.002–27.566 µM	28.523 µM 0.002 µM	2019	[153]
Diazinon	Fluorescence	UCNPs/Cu^2+^- AChE	0.033–164.285 nM	0.164 nM	2019	[197]
Demeton	Fluorescence	QDs-nanoporphyrin	38.709–77.418 nM	38.709 nM	2019	[139]
Chlorpyrifos	LC–MS/MS	AChE	-	37.080 nM	2020	[138]
Methyl parathion	SERS	silver nanoparticles-Al_2_O_3_	-	1.000 fM	2020	[178]
Chlorpyrifos	SERS	silver nitrate	1.000 mM–1.000 nM	1.000 nM	2020	[191]
Methyl parathion	SERS	AuNPs	-	0.004 µM	2020	[179]
Fenirothion	SPR	tantalum(V) oxide nanoparticles	0.250–4.000 μM	0.038 µM	2020	[203]

where LOD is limit of detection. AIE: aggregation induced emission, Al_2_O_3_: aluminum oxide, ATCH: acetylthiocholine iodide, BiFeO_3_: bismuth ferrit nanocomposites, CdS: cadmium sulfide, CdSe: cadmium selenide, CdTe: cadmium tellurite, cFLISA: competitive fluorescence-linked immunosorbent assay, CTAB: cetyltrimethylammonium bromide, CuInS_2_: copper indium sulfide, GAA: guanidineacetic acid, g-C_3_N_4_: graphitic carbon nitrite, HQO: 2-hydroyquinoxaline, LC-MS/MS: liquid chromatography–mass spectrometry, LSPR: localized surface plasmon resonance, Lum-AgNP: luminol-functionalized silver nanoparticle, MAbs: monoclonal antibody, MPA: 3-mercaptopropinonic acid, MPDE: methyl parathion degrading enzyme, MWCNT: multi-walled carbon nanotubes, NCs: nanocrystal, PAH: poly(allylamine hydrochloride), TCP: 3, 5, 6-trichloropyridid, TPE: tetraphenylethylene, TPPS4: tetrakis(4-sulfonatophenyl)porphyrin, UCNPs: upconventional nanoparticles, ZnSe: zinc selenide, ZnS: zinc sulfide.

**Table 4:** Chronological order of optical sensors for phosphorothioates (s-substituted) detection.

Type of Phosphorothioates (S-Substituted)	Method	Material	Range of Detection	LOD	Year	References
Omethoate	Colorimetric	indoxyl acetate-R-DmAChE	4.691–46.907 µM	29.551 µM	2012	[137]
Profenofos	SPR	SAM/ 2,2-azobis (2-amidinopropane) hydrochloride	0.003–0.268 nM	0.964 pM	2012	[213]
Omethoate	Fluorescence	gold-based nanobeacon	0.268–26.800 µM	2.350 µM	2015	[210]
Profenofos	SPR	MIPs-Ag	-	0.007 nM	2016	[212]
Profenofos	Fluorescence	CdTe/CdS-QDs (AMO-aptamer)	-	0.100 µM 0.230 µM	2016	[214]
Omethoate						
Malaoxon	Fluorescence	QDs-AChE/ChOx	-	0.050 µM	2017	[136]
Omethoate	Colorimetric	silver metallization of AuNRs-ALP	-	0.390 nM	2020	[211]
Profenofos	Luminescent	Eu-IRMOF-3-EBA	-	0.002 nM	2020	[215]
Azamethiphos	Fluorescence	8-((E)-((thiophen-2-yl)methylimino)methyl)-7-hydroxy-4-methyl-2H-chromen-2-one (L) to copper (II) ion	0–50.000 µM	50.000 µM	2020	[216]

where LOD is limit of detection. AuNRs: gold nanorods, AMO: amino-modified oligonucleotide, CdSQDs: cadmium sulfide quantum dots, CdTe: cadmium tellurite, EBA: ethylbenzoylacetate, Eu: europium, MOF: metal organic framework, R-DmAChE: recombinant Drosophila melanogaster acetylcholinesterase.

**Table 5:** Chronological order of optical sensors for phosphorodithioates detection.

Type of Phosphorodithioates	Method	Material	Range of Detection	LOD	Year	References
Malathion	Colorimetric	IPA/sol-gel derived silica inks-AChE	0–10.000 µM	0.001 µM	2009	[121]
Malathion	SERS	MCR-WALS	0.372–37.232 µM	0.372 µM	2015	[231]
Malathion	Colorimetric	dithiobis-nitrobenzoic acid-AChE	0–24.216 µM	0.303 µM	2015	[229]
Dimethoate	Fluorescence	NaYF4:Yb,Er/UCNPs-AChE	0.009–87.237 nM	0.292 nM	2015	[148]
Malathion	Colorimetric	gold nanorods- HRP	0.003–1.816 mM	0.005 mM	2016	[230]
Malathion	Colorimetric	palladium-gold	0.003–605.404 nM	181.621 nM	2017	[232]
Malathion	Fluorescence	QDs-AChE/ChOx	0.001–0.100 µM	0.05 µM	2017	[137]
Phosmet	Fluorescence	PDs/Ab based poly [2-methoxy-5-(2-ethylhexyloxy)-1, 4-(1-cyanovinylene-1, 4-phenylene)]	0.007–0.126 nM	0.126 nM	2017	[233]
Carbophenothion	SERS	AuNPs	0.003–29.167 µM	0.292 µM	2019	[153]
Malathion			0.003–30.270 µM	0.327 µM		
Phosalone			0.003–27.188 µM	2.717 µM		
Phosmet			0.003–31.515 µM	0.315 µM		
Dimethoate	Fluorescence	QDs-nanoporphyrin	0.044–0.630 µM	0.044 µM	2019	[139]
Dimethoate	SPR	P(EGDMA-MATrp)	0.040–4.360 nM	0.033 nM	2019	[234]
Ethion	Luminescent	Eu-IRMOF-3-ethylbenzoylacetate	-	0.003 nM	2020	[215]

where LOD is limit of detection. DMOAP: dimethyloctadecyl[3-(trimethoxysilyl)propyl]ammonium chloride, Eu: europium, GNRs: gold nanorods, GOPs: (3-glycidyloxypropyl)trimethoxysilane, IPA: idophenyl acetate, LC: liquid crystal, LSPR: localized surface plasmon resonance, MCR-WALS: multivariate curve calibration-weighted alternating least square, MOF: metal organic framework, PDDA: polyelectrolyte polydiallydimethylammonium chloride, PDs: polymer dots, UCNPs: upconventional nanoparticles.

**Table 6:** Chronological order of optical sensors for phosphoramidates detection.

Type of Phosphoramidates	Method	Material	Range of Detection	LOD	Year	References
Fenamithion	Luminescence	CdSeQDs -p-sulfonatocalix[4]arene	0–100.000 µM	0.012 µM	2009	[237]
Fenamithion	Fluorescence and colorimetric	RB-AgNPs	-	10.000 nM	2011	[238]
Fenamiphos	Colorimetric	AuNPs	-	0.247 µM	2016	[202]

where LOD is limit of detection. AuNPs: gold nanoparticles, CdSeQDs: cadmium tellurite quantum dots, RB-AgNPs: rhodamine-silver nanoparticles.

**Table 7:** Chronological order of optical sensors for phosphoramidothioates detection.

Type of Phosphoramidothioates	Method	Material	Range of Detection	LOD	Year	References
Methamidophos	Colorimetric	indoxyl acetate-R-DmAChE	-	223.955 µM	2012	[136]
Acephate	Colorimetric	Fe_3_O_4_(MNP)-AChE/ChOx	-	0.005 mM	2013	[140]
Isocarbophos	LFIA	monoclonal antibody	0.346–20.740 µM	0.346 µM	2014	[201]
Isocarbophos	Fluorescence	gold-based nanobeacon	-	0.035 µM	2015	[210]
Acephate	Fluorescence	1, 8-naphthalimide-AChE/ChOx	-	0.006 nM	2016	[152]
Isocarbophos	Fluorescence	CdTe/CdS-QDs(aptamer)	-	0.170 µM	2016	[214]

where LOD is limit of detection. CdS: cadmium sulfide, CdTe: cadmium tellurite, MNP: magnetic nanoparticle, R-DmAChE: recombinant Drosophila melanogaster acetylcholinesterase.

**Table 8:** Chronological order of optical sensor for phosphonofluoridates detection.

Type of Phosphonofluoridates	Method	Material	Range of Detection	LOD	Year	References
**Sarin**	Colorimetric	LA-AUNPs-AChE	28.200–225.000 pM	28.200 pM	2011	[246]
**Sarin**	Colorimetric	Fe_2_O_3_-MNPs-AChE	-	1.000 nM	2013	[140]

where LOD is limit of detection. Fe_2_O_3_: iron(III) oxide, MNPs: magnetic nanoparticles, LA: lipoic acid.

**Table 9:** Chronological order of optical sensors for carbamates detection.

Type of Carbamates	Method	Material	Range of Detection	LOD	Year	References
Carbaryl	SPR	SAM/mAbs	-	6.858 mM	2005	[258]
Carbaryl	SPR	mAbs	0.089–0.268 nM	0.248 nM	2007	[185]
Methomyl	Fluorescence	C[4]/SiO_2_/CdTe	-	0.080 µM	2007	[256]
Carbofuran	LFIA	anti-carbofuran	0–128.000 µM	32.000 µM	2009	[257]
Trizophos		anti-trizophos	0–32.000 µM	4.000 µM		
Bendiocarb	Colorimetric	idophenyl acetate-AChE	0–10.000 nM	0.001 µM	2009	[121]
Carbaryl				10.000 nM		
Carbofuran	Colorimetric	indoxyl acetate-R-DmAChE	0.005–45.197 µM0.006–61.648 µM	27.118 µM	2012	[137]
Methomyl				1.726 µM		
Carbaryl	Photoluminescence	SiQDs-AChE/ChOx	0.037–3722.29 nM	0.037 nM	2013	[195]
Carbaryl	Colorimetric	PABSA-AuNPs	0.100 nM–1 mM	0.250 µM	2013	[259]
Carbaryl	Fluorescence	CdSe/ZnSQDs	-	0.147 µM	2015	[260]
Metolcarb	Fluorescence	NOC4	0.100 nM–1.000 mM	0.100 µM	2015	[265]
Carbaryl	Chemiluminescent	Lum-AgNP	0–4.970 µM	4.970 µM	2015	[151]
			0–108.472 µM	108.472 µM		
Carbofuran						
Carbaryl	Colorimetric and chemiluminescent	dual-g-C_3_N_4_/BiFeO_3_	0.005–0.298 µM	0.164 nM	2018	[192]
Carbofuran	SERS	AuNPs	0.005–45.197 µM	0.904 µM	2019	[153]
			0.006–61.648 µM	0.123 µM		
Methomyl						
Carbendazim	SERS	AuNPs	0–52.305 µM	0.523 µM	2019	[266]
Carbofuran	SPR	P(EGDMA-MATrp)	-	0.032 nM	2019	[234]
Carbendazim	SPR	AuNPs- Fe_3_O_4_/mAbs	0.262–784.572 nM	2.301 nM	2019	[267]
Aldicarb Carbofuran Carbofuran-3 hydroxy Carbaryl	Liquid Chromatography Tandem Mass Spectrometry	AChE	-	0.039 µM	2020	[138]
			-	0.007 µM		
			-	5.901 pM		
			-	0.007 µM		
Carbaryl	HFF-QCM	mAbs	0.497 pM–4.970 nM	0.248 nM	2020	[261]
Carbaryl	Fluorescence	CdTeQDs	-	0.596 nM	2020	[262]
Carbaryl	Fluorescence	CQDs-AuNPs-AChE	0.994–745.453 nM	0.298 nM	2020	[263]
Carbaryl	Colorimetric	silver reduced-graphene oxide	0.100–50.000 μM	42.000 nM	2020	[264]

where LOD is limit of detection. CdSe: cadmium selenide, c[4]/SiO_2_/CdTe: 5, 11, 17, 23-tetra-tert-butyl-25, 27-diethoxy-26, 28-dihydroxycalix[4]arene/ silicon dioxide/cadmium tellurite, g-C_3_N_4_/BiFeO_3_: graphitic carbon nitrite/bismuth ferrite nanocomposite, HFF-QCM: high fundamental frequency quartz crystal microbalance, LFIA: lateral flow immunoassay, Lum-AgNP: luminol-functionalized silver nanoparticles, NOC4: naphthol-appended calix[4]arene, P(EGDMA-MATrp): ethylene glycol dimetacrylate-N-metacryloyl-(l)-tryptophan methyl ester-p, R-DMAChE: recombinant Drosophila melanogaster acetylcholinesterase, Re(I)-NCS-Pt(II): [Re(4,4′-di-tert-butyl-2,2′-bupryridine)(CO)_3_(NCS)], SERS: surface-enhanced Raman spectroscopy, SiQDs: silicon quantum dots, PABSA: 4-acetamidobenzenesulfonyl azide, ZnSQDs: zinc selunide quantum dots.

**Table 10:** Chronological order of optical sensors for neonicotinoids detection.

Type of Neonicotinoids	Method	Material	Range of Detection	LOD	Year	References
Acetamiprid	Luminescence	CdTeQDs and p sulfonatocalix[4]arene	0–1000.000 µM	0.034 µM	2009	[237]
Acetamiprid	Colorimetric	AuNPs	0.660–6.600 µM	0.044 µM	2011	[277]
Acetamiprid	Fluorescence	CdSe/ZnSe/ZnS QDs-AChE	0.225–44.910 nM	4.491 nM	2013	[283]
Acetamiprid	Colorimetric	AuNPs-ABA	0.075–7.500 µM	0.005 µM	2013	[278]
Acetamiprid	Colorimetric	AuNPs	0.449–44.910 µM	0.449 µM	2014	[273]
Thiacloprid	ELISA	Anti-thiacloprid	0–0.723 µM	0.017 µM	2014	[285]
Imidacloprid		Anti-imidacloprid	0–0.228 µM	0.008 µM		
Acetamiprid	Fluorescence	AuNPs	0.112–22.455 µM	75.448 µM	2014	[279]
Imidacloprid	Chemiluminescence	bispecific monoclonal antibody-HRP/ALP	-	0.001 µM	2015	[172]
Acetamiprid	Colorimetric	hemin-reduced graphene oxide	0.100–10.000 µM	40.000 nM	2015	[274]
Acetamiprid	Fluorescence	NH_2_-NaYF_4_: Yb, Ho@SiO_2_/UCNPs/GNPs	-	0.003 µM	2016	[275]
Acetamiprid	Fluorescence	ZnS:Mn-aptamer and MWCNTs	0–150.000 nM	0.700 nM	2016	[276]
Acetamiprid	Fluorescence	AuNPs/SWNTs/SiNPs-streptavidin	0–1000.000 nM	127.000 pM	2016	[284]
Imidacloprid	Colorimetric	AuNPs-AChE	0.156–1.565 µM	0.939 µM	2016	[202]
Acetamiprid	Chemiluminescence	AuNPs	0.800 nM–0.630 µM	62.000 pM	2016	[280]
Acetamiprid	Colorimetric	AuNPs	0–50.000 µM	0.400 µM	2016	[281]
Imidacloprid	Chemiluminescent	bispecific antibody-HRP/ALP	-	0.227 nM	2017	[173]
Acetamiprid	Colorimetric	AuNPs	-	0.560 nM	2020	[282]
Imidacloprid	LFIA	monoclonal antibody	0–78.229 pM	78.229 pM	2020	[286]
Imidacloprid	SERS	AgNPs	0–0.110 nM	0.110 nM	2020	[287]

where LOD is limit of detection. CdSe: cadmium telluride, CdTe: cadmium selenide, ELISA: enzyme-linked immunosorbent assay, LFIA: lateral flow immunoassay, Mn: manganese: MWCNTs: multi-walled carbon nanotubes, SiO_2_: silicon dioxide, UCNPs: upconventional nanoparticles, ZnS: zinc sulfide, ZnSe: zinc selenide.

**Table 11:** Chronological order of optical sensors for pyrethroids/pyrethrins detection.

Type of Pyrethroids/Pyrethrins	Method	Material	Range of Detection	LOD	Year	References
Cyhalothrin	Fluorescence	MIPs-CdSe/SiO_2_	0.100–1000.000 µM	0.101 µM	2010	[294]
Cyphenothrin	Fluorescence	MIPs-Mn doped ZnS QDs	0.100–80.000 µM	9.000 µM	2014	[297]
Cypermethrin	ELISA	MIPs-QDs	-	0.003 µM	2015	[298]
Cyhalothrin	Fluorescence	SiO_2_-MIPs	0–1.500 µM	10.260 nM	2016	[295]
Cyhalothrin	Fluorescence	OVDAC/CdTeQDs	0.100–16.000 µM	0.030 µM	2016	[296]
Permethrin	SERS	AuNPs	0.002–25.557 µM	0.002 µM	2019	[153]
Transfluthrin			0.002–26.943 µM	2.694 µM		

where LOD is limit of detection. CdSe: cadmium telluride, CdTe: cadmium selenide, ELISA: enzyme-linked immunosorbent assay, Mn: manganese, MIP: molecular imprinted polymer, OVDAC: octadecyl-4-vinylbenzyl-dimethytammonium, SiO_2_: silicon dioxide.

**Table 12:** Chronological order of optical sensors for organochlorines detection.

Type of Organochlorines	Methods	Materials	LOD	Year	References
DDT	SPR	anti-DDT monoclonal antibody (LIB-DDT5.25)	0.141 nM	2007	[185]
Dieldrin	SERS	alkyl dithiol-functionalized metal nanoparticles-induced plasmonic hot spots	0.123 µM	2015	[306]
Aldrin			0.418 µM		
Endosulfan			3.534 µM		
Lindane			0.830 µM		
DDT	HFF-QCM	anti-DDT monoclonal antibody	0.068 nM	2020	[261]

where LOD is limit of detection. HFF-QCM: high fundamental frequency quartz crystal microbalance, SERS: surface enhanced Raman scattering, SPR: surface plasmon resonance.

**Table 13:** Insecticide detection based on optical sensors with the lowest LOD.

Optical Method	Insecticides	Materials	LOD	References
Fluorescence	Dichlorvos	CdTeQDs-AChE	0.0021 nM	[134]
Colorimetric	Sarin	lipoic acid-AuNPs-AChE	0.0282 nM	[246]
SERS	Imidacloprid	AgNPs	0.1100 nM	[287]
SPR	Carbofuran	P(EGDMA-MATrp)	0.0320 nM	[234]
Chemiluminescence	Acetamiprid	AuNPs	0.0620 nM	[280]

where LOD is limit of detection. Al_2_O_3_: aluminum oxide, AChE: acetylcholinesterase, AgNPs: silver nanoparticles, AuNPs: gold nanoparticles, CdTeQDs: cadmium telurrite quantum dots, P(EGDMA-MATrp): ethylene glycol dimetacrylate-N-metacryloyl-(l)-tryptophan methyl ester-p, SERS: surface enhanced Raman scattering, SPR: surface plasmon resonance.
